# The primacy model and the structure of olfactory space

**DOI:** 10.1371/journal.pcbi.1012379

**Published:** 2024-09-10

**Authors:** Hamza Giaffar, Sergey Shuvaev, Dmitry Rinberg, Alexei A. Koulakov

**Affiliations:** 1 Cold Spring Harbor Laboratory, Cold Spring Harbor, New York, United States of America; 2 Neuroscience Institute, New York University Langone Health, New York, New York, United States of America; 3 Center for Neural Science, New York University, New York, New York, United States of America; University of California Santa Barbara, UNITED STATES OF AMERICA

## Abstract

Understanding sensory processing involves relating the stimulus space, its neural representation, and perceptual quality. In olfaction, the difficulty in establishing these links lies partly in the complexity of the underlying odor input space and perceptual responses. Based on the recently proposed primacy model for concentration invariant odor identity representation and a few assumptions, we have developed a theoretical framework for mapping the odor input space to the response properties of olfactory receptors. We analyze a geometrical structure containing odor representations in a multidimensional space of receptor affinities and describe its low-dimensional implementation, the primacy hull. We propose the implications of the primacy hull for the structure of feedforward connectivity in early olfactory networks. We test the predictions of our theory by comparing the existing receptor-ligand affinity and connectivity data obtained in the fruit fly olfactory system. We find that the Kenyon cells of the insect mushroom body integrate inputs from the high-affinity (primacy) sets of olfactory receptors in agreement with the primacy theory.

## Author summary

Sensory systems face the problem of computing stimulus identity that is invariant with respect to multiple stimulus features. In the olfactory system, odorant percepts often retain their identity despite substantial variations in the odorant concentration. How can olfactory networks robustly represent odorant identity despite variable stimulus intensity? In the nose, odorants are sensed by the odorant receptors (ORs), the specialized proteins that can be activated by the odorant molecules. In this study, we develop a theory for encoding concentration-invariant odor objects. We propose that the identities of a small group of the most sensitive ORs represent odorant identity in a concentration-invariant manner. We argue that, although absolute values of olfactory receptor responses may depend on concentration, their ranking remains invariant, leading to invariance in the membership of the most sensitive (primary) group. We call this form of representation of concentration invariant odorant identity the *primacy coding model*. We investigate the mathematical principles underlying the primacy coding model and test its predictions using connectivity and neuronal activations data from the fruit fly olfactory system. We find that neurons in the fly olfactory system integrate inputs from the high-affinity (primacy) sets of ORs in agreement with the primacy theory.

## Introduction

Our ability to predict the perceptual quality of color from a spectrum of incident light relies on a small number of receptor types at the neural periphery and our understanding of the properties of these receptors. The dimensionality of the color space is defined by the three types of receptors, i.e., three degrees of freedom, two of which define the planar coordinates of the color and one that computes the total intensity of the light. The olfactory system operates with a much larger number of receptor types at the sensory periphery (~350 in humans, ~1200 in rodents, and ~60 in flies [1–5]). The mapping of the chemical stimulus space to the receptor and perceptual spaces remains an unresolved problem. The discovery of such a large family of olfactory receptors (ORs) [6] has prompted the idea that the dimensionality, *D*, of this olfactory space is high and comparable to the number of OR types, *N*.

There is emerging evidence, however, that olfactory perceptual space is not so high dimensional [7,8]. The embedding of human perceptual data into a curved manifold of dimension *D*<10 accounts for >80% of the variance in the data, suggesting that the number of odorant parameters relevant to the human olfactory system is <10 [8–10]. A recent success in predicting olfactory metamers for humans using a mixture model, which describes each odorant with ~20 parameters, also suggests low dimensionality of the odor perceptual space [11]. Insights into the structure and dimensionality of olfactory perceptual space should guide our understanding of information processing in this sensory system.

Several features of early olfactory neural circuits are strongly conserved across diverse species, from insects to mammals [12]. Olfactory sensory neurons (OSNs) expressing ORs of the same genetic type converge on their respective glomeruli in the vertebrate olfactory bulb (OB) or antennal lobe (AL) in insects. To a first approximation, each glomerulus represents the level of activation of a single OR type. This information is projected to several higher-level processing centers, such as the Piriform Cortex (PC) in mammals or the Mushroom Body (MB) in insects, by axons of the second order projection neurons. The logic underlying the connectivity between the OB/AL and these downstream target areas has been under intense scrutiny as it seems to convey the nature of the features important for olfactory processing. The convergent evolution of this ‘canonical’ olfactory circuit may suggest a conserved logic of odor information processing across phyla [13]. If this is true, it should be possible to construct a general theory of olfactory information processing relevant to a wide range of species.

As in the case of vision, where a color percept is formed from light with a broad spectrum of monochromatic waves, a majority of ethologically relevant odors are mixtures of many tens or hundreds of components [14,15]. Humans often perceive complex mixtures synthetically, identifying a single ‘odor object’ rather than recognizing the individual components [16]. In addition, the perceptual identity of an odor is generally stable over a range of both stimulus and neural parameters, including concentration, background and noise in neural circuits [17–21]. This feature of the neural code may contribute to the ability of animals to identify sources of smells in variable environments and at varying distances.

How does an odorant retain its perceptual identity despite changes in concentration? As an odorant concentration increases from low to high, more OR types generally become activated [22–24]. The representations of odor identity in high and low concentration regimes can therefore be linked by a set of ORs that activated at low concentration and are active in both regimes. This template comprised of high affinity OR types to a given odorant is called the *primacy set* and the model for concentration-invariant odor coding relying on primacy sets is called the *primacy model* [25]. Despite its apparent simplicity, the primacy model can explain many psychophysical phenomena of odor perception and is compatible with known olfactory neural network organization [25–28].

Here, we propose a new theoretical framework for mapping olfactory chemical space to the neural spaces of OR/glomerular and higher order representations. Our theory is based on the following main assumptions: (i) The stimulus or, in other words, odor space is of relatively low intrinsic dimensionality. (ii) Odor identity is encoded by a small number of OR types of highest affinity for a given odor (*primacy coding hypothesis*). We will study the implications of these assumptions for the evolution of OR ensembles and develop statistical methods to test these assumptions in recently published connectivity data from the fly olfactory system.

## Results

### Primacy coding model

We will consider a model in which the activation of a receptor *f*_*r*_, as a function of odorant concentration, *c*_*o*_, depends only on one parameter *K*_*ro*_, the affinity of receptor *r* to odorant *o*, and can be described by the mass action law:

$$\frac{f_{r}}{1-f_{r}}=K_{ro}c_{o}$$ (1)

**Fig 1:**
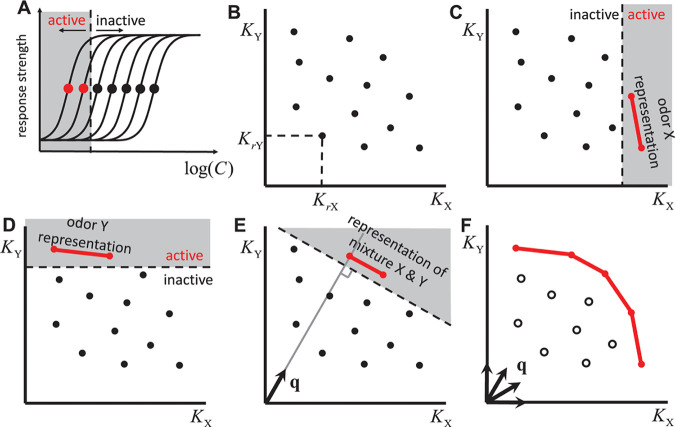
Primacy model in 2D. **A.** Receptor response curves as a function of odorant concentration. Solid circles correspond to effective threshold concentrations or inverse affinities: $c_{ro}^{th}=(K_{ro}{)}^{-1}$. For a certain concentration (dashed line), the ORs with a threshold below this concentration (solid red circles) are in an active state. These ORs form a primacy set with the primacy number *p* = 2 for this odorant. **B.** Representation of receptors in 2D space of affinities for odorants *X* and *Y*: *K*_*X*_ and *K*_*Y*_. **C.** An odorant *X* at concentration *c*_*X*_ activates all receptors for which *K*_*rX*_*c*_*X*_>1 (red). For the given primacy number *p* = 2, the identity of odor *X* is defined by the two most sensitive receptors (red segment). **D.** The same for odor *Y*. **E.** A mixture of two odorants *X* and *Y* activates receptors, which are above the line perpendicular to the unit vector ***q*** = [*c*_*X*_, *c*_*Y*_]/*c*, where $c=\sqrt{c_{X}^{2}+c_{Y}^{2}}$. **F.** Primacy sets for all possible mixture vectors ***q*** define a *primacy hull* (red), a 1D line in the 2D space. Receptors in the primacy hull (red) are retained in the genome. All other ORs (empty circles) are expected to be eliminated from the genome (pseudogenized).

In logarithmic concentration coordinates, *f*_*r*_ = *f*_*r*_(*c*_*o*_) is a logistic function (Fig 1A) [23]. When the activation of the receptor reaches a certain threshold, *θ*, the changes in the response can be detected by the downstream system, at which point, the OR becomes activated and can participate in the odorant coding. For simplicity, we will define an OR as active if its response to an odorant is higher than a half of the maximum activity level, i.e., *f*_*r*_>*θ* = 1/2, which corresponds to the odorant concentration *c*_*o*_>1/*K*_*ro*_. Our conclusions are not affected by the choice of activation threshold, as long as it is similar across all receptors. Importantly, in this model, receptors that are activated at the lowest concentration remain active at higher concentrations.

According to the primacy coding hypothesis, the identities of a few of the most sensitive OR types determine the perceptual odor identity associated with a stimulus. We define the primacy set for a given odorant as the set of *p* OR types activated by the odorant at the lowest concentration (Fig 1A). Different odorants evoke activity in different sets of most sensitive receptors. The primacy number, *p*, could vary across odorants; however, here we will assume it to be fixed for simplicity.

### A two-dimensional odor space

To explore the implications of primacy coding for the organization of an OR ensemble, we consider an odor space comprised of two odorants (*X*, *Y*) and their mixtures. In this case, each OR type can be represented as a point in the 2D space of affinities for the two odorants with coordinates ***K***_*r*_ = (*K*_*rX*_, *K*_*rY*_). An example arrangement of a receptor ensemble in a 2D odor space is shown in Fig 1B. Introducing a pure odorant *X* at a concentration *c*_*X*_ partitions the space into two half spaces; one in which receptors are active, *K*_*rX*_*c*_*X*_>1, and the other in which they are inactive, *K*_*rX*_*c*_*X*_<1 (Fig 1C). Increasing *c*_*X*_ moves the boundary between the active and inactive receptors and expands the zone of active receptors from high to low *K*_*X*_. If we consider a primacy model in which two glomeruli are required to identify an odor (a *p* = 2 primacy model), there is a concentration of odor *X* at which the first two glomeruli are activated. These glomeruli represent OR types of highest affinity for odorant *X* and represent the identity of *X* in the primacy coding mechanism (Fig 1C).

Similarly, the primacy set corresponding to an odor *Y* can be identified by introducing the pure odor *Y* at a low concentration and expanding the zone of the active ORs by increasing the concentration until the first *p* = 2 OR types are activated (Fig 1D).

Eq (1) can be extended to the case of two or more odorants. Assuming the independence of individual odorant-receptor interactions, we have (Methods C in S1 Text):

$$\frac{f_{r}}{1-f_{r}}=K_{rX}c_{X}+K_{rY}c_{Y}=(K_{r}\cdot c).$$ (2)

Here (***K***_*r*_·***c***) is the scalar product between the vector of affinities of a receptor *r* for the set of molecules *X* and *Y*, ***K***_*r*_ = (*K*_*rX*_, *K*_*rY*_), and the vector of concentrations ***c*** = (*c*_*X*_, *c*_*Y*_). Receptor activations can be described by an equation that explicitly accounts for the overall concentration of the odor mixture:

$$\frac{f_{r}}{1-f_{r}}=(K_{r}\cdot q)c$$ (3)

Here $c=\sqrt{c_{X}^{2}+c_{Y}^{2}}$ is the Euclidian length of the concentration vector representing the overall mixture concentration and ***q*** = [*c*_*X*_, *c*_*Y*_]/*c* is a unit vector in the direction defined by the ratio of the mixture components’ concentrations. For a given mixture concentration *c*, the activation level of an OR is determined by the projection of its affinity vector ***K***_*r*_ and the unit vector describing the concentrations of the mixture components ***q***, i.e. ***K***_*r*_·***q***. The most active ORs are those with the largest value of ***K***_*r*_·***q*** (Fig 1E). For a *p* = 2 primacy model, the primary receptors are the two points with the largest projection onto the vector ***q***, i.e. the two ORs activated at the lowest concentration of the mixture (Fig 1E). By considering the primacy sets of all possible mixtures, i.e. all possible vectors ***q*** in this 2D odor space, we trace out a hull containing all OR types that belong to at least one primary set (Fig 1F). We call this structure a ***primacy hull***.

The primacy hull contains all OR types that belong to at least one primacy set. According to the primacy model defined here, odor identity is encoded by the OR types of highest affinity for a given odorant, i.e., primary ORs. The primacy hull includes *all* odorant identities that can be encoded by a particular set of ORs within the primacy coding model (four in Fig 1F). ORs that have low affinities for every odorant are not included in any primacy set and do not participate in odor identity coding. Unless such ORs participate in the encoding of odorant features other than identity or have some other non-olfactory coding function[13], they are likely to be pseudogenized and eliminated from the genome (Fig 1F). Thus, one of the predictions of the primacy model is that the functional ORs should belong to a primacy set of at least one odorant and therefore belong to the primacy hull, unless they are involved in some other processes.

Eqs (1–3) follow from the receptor-ligand binding model which does not include some important mechanisms involved in odorant-OR interactions, such as antagonism [29, 30] or potential multiple odorant binding sites per OR [31]. Nevertheless, following Refs. [31–37], we will adopt this model for mathematical convenience. Importantly, in the linear model Eqs (1–3), primacy sets do not depend on the choice of activation threshold, as long as it is the same for all receptor-odorant pairs. Indeed, according to Eq (3), primary receptors can be determined as *p* ORs with the highest projection of the affinity vector ***K***_*r*_ on the mixture concentration vector ***q***, i.e. the receptors activated at the lowest mixture concentration. This statement is not affected by the particular response level *f*_*r*_ = *θ* at which this response becomes detectable. We therefore adopted *θ* = 1/2 here for simplicity. We analyze the implications of nonlinear interactions between odorant molecules for the primacy model in Methods D in S1 Text.

### Higher-dimensional odor spaces

**Fig 2:**
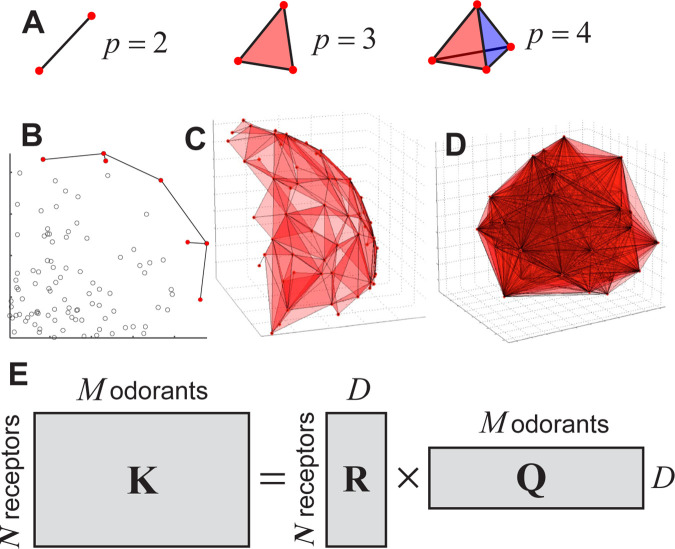
Primacy hull in higher dimensions. **A.** Geometrical representation of primacy sets for different primacy numbers: *p* = 2: 1-simplex, a segment; *p* = 3: 2-simplex, a triangle; *p* = 4: 3-simplex, tetrahedron. **B**. An example of primacy hull for a random set of points in 2D (*p* = 2). A primacy hull is a set of simplexes that reside on the extremes of the given set of points. **C.** A primacy hull for *D* = 3, *p* = 3. A ~2D surface is tessellated by triangles, each of them representing an independent odor identity. **D.** The same for *D* = 6, *p* = 7. 6D manifold is projected onto 3D space for visualization. **E.** Decomposition of affinity matrix ***K*** into two low-dimensional matrices: ***Q*** is a low-dimensional matrix of basic odor features and ***R*** is a matrix of receptor affinities for these basic features.

As in the preceding 2D model, in the case of more odorants present, ORs can be represented by vectors of affinities to individual odorants in a D-dimensional odor space: ***K***_*r*_ = [*K*_*r*1_, *K*_*r*2_,…,*K*_*rD*_]. The receptor response to a mixture is then described by Eq (3) with the odor mixture represented by the unit vector: ***q*** = [*c*_1_, *c*_2_,…,*c*_*D*_]/*c* where $c=(c_{1}^{2}+c_{2}^{2}+\ldots +c_{D}^{2}{)}^{1/2}$. At a given concentration, a *D*−1-dimensional plane orthogonal to the vector ***q*** separates the active and inactive receptors. Increasing the odor concentration moves this plane toward the origin of the coordinate system and recruits additional receptors. The first *p* activated ORs form a primacy set for the mixture defined by the vector ***q***. The primacy number, *p*, and the dimensionality of the odor space, *D*, are independent parameters. In the previous example we explored the case in which *p* = 2; each odor identity is represented by two nodes (OR types), which may be thought of as a segment. In general, a primacy set can be represented by a composition of *p* interconnected points forming a (*p*−1) -simplex: *p* = 2 corresponds to a line segment, *p* = 3 forms a triangle, and *p* = 4 forms a tetrahedron (Fig 2A).

The primacy hull is a simplicial complex (a collection of simplexes) obtained by sweeping planes through a collection of points; for each plane, the first *p* encountered points (those of the highest affinity) are associated into a simplex and added to the complex (Fig 1). The primacy hull therefore includes the convex hull as a subset; it may also include points internal to the convex hull (Fig 2B). Examples of primacy hulls for *D* = 3, *p* = 3 and for *D* = 6, *p* = 7 are shown in Fig 2C and 2D. The latter hull is projected onto 3D space for display purposes.

The primacy hull includes *all* odorant identities that can be encoded by a particular set of ORs within the primacy coding model. Its vertices include the ORs that belong to at least one primacy set. Its edges connect ORs that belong to the same primacy set. Odorant mixtures of similar composition are expected to yield the same primacy set if the corresponding q-vectors are close to each other in the odor space. The number of odorant identities in the primacy hull is finite but grows both with the number of ORs and the primacy number. We assume that the number of odorant identities encoded using this mechanism is sufficient to represents and distinguish the set of relevant mixtures.

The full set of volatile molecules likely includes millions of compounds, yet the description of a primacy hull in *D*~10^6^ is not very useful. As discussed in the introduction, some experimental evidence suggests that the dimensionality of the odor space is low. We will next formulate the low-dimensionality assumption within our model. Eq (2) relates receptor activity to both receptor affinities and concentrations of mixture components. The affinities can be determined as the inverse concentration thresholds $K_{ro}=(c_{ro}^{\mathrm{th}}{)}^{-1}$ estimated from the concentration dependencies of neural responses (Fig 1A). Here $c_{ro}^{\mathrm{th}}$ is the concentration of a monomolecular odorant *o*, at which a receptor *r* is activated at half of its maximum magnitude.

The affinities *K*_*ro*_ used in Eq (2) describe the affinity of *N* ORs for *M* monomolecular odorants. These quantities can be combined into an affinity matrix ***K*** which has dimensions *N*×*M* (*N*~10^3^, *M*~10^7^). If ***K*** can be accurately represented by a product of two matrices of much smaller size, ***R***_*N*×*D*_ and ***Q***_*D*×*M*_, where *D*≪*N*, *M*, we can say that the dimensionality of the odor space is low (Fig 2E):

K=R·Q (4)

Here ***Q*** is a *D*×*M* matrix placing every molecule into a D-dimensional space of “properties of interest” to the olfactory system. It describes to what degree these properties are present in each of the *M* molecules. These properties may include molecular weight, measures of polarity, size, and other potentially more complex molecular features [8, 10, 38]. ***R*** is a *N*×*D* matrix representing every receptor as a point in the D-dimensional space of molecular properties. The affinity of the receptor *r* for a (monomolecular) odorant *o* is determined by the scalar product of the corresponding row in the matrix ***R*** and a column in the matrix ***Q***, both of which are D-dimensional. If the number of odorant parameters sampled by the olfactory system is on the order of the dimensionality of human perceptual space, i.e. *D*~10, then the simplification resulting from Eq (4) can be substantial [8, 10].

Eq (4) describes the affinities of receptors to pure odorants. The responses to mixtures can be obtained by combining Eqs (2) and (4), which results in

$$\frac{f_{r}}{1-f_{r}}=(R_{r}\cdot \tilde{q})$$ (5)

Here, we introduced a D-dimensional vector $\tilde{q}=Qc$ describing the concentration of odorant properties in the mixture described by the vector ***c***. For example, if the ORs were sensitive to the molecular weights of monomolecular compounds, as suggested in Ref. [10], one component of the vector $\tilde{q}$ would represent the concentration of molecular weight, i.e. the molecular weight per liter of gas. The role of the *D*×*M* property matrix ***Q*** is therefore to project the concentration vectors of mixture components which may have millions of dimensions to a much smaller *D* -dimensional space of properties relevant to the olfactory system. The vector ***R***_*r*_ is a *D* -dimensional row of the matrix ***R*** which describes the OR sensitivity to these properties. In the case of mixtures, the activation level of a receptor is determined by the dot product between the low-dimensional vectors of affinities and property concentrations, similarly to Eq (2). In this case, instead of a description in the space of affinity vectors ***K***_*r*_, the primacy sets and the primacy hull are built in the space of relevant odorant properties ***R***_*r*_. This implies that diagrams like the one presented in Figs 1 and 2B–2D are constructed from the components of vectors ***R***_*r*_ rather than vectors ***K***_*r*_. Thus, the approach described above, including the geometric constructs, such as primacy sets and the primacy hull, is valid in the case of low-dimensional olfactory space [Eq (4)] despite the odorant mixtures including, potentially, millions of monomolecular components. Overall, we suggest that, in the case of low-dimensional odor space, the mechanism of coding of odor identity in the primacy sets described above applies in the space of odorant properties.

#### Connectivity to higher brain regions

**Fig 3:**
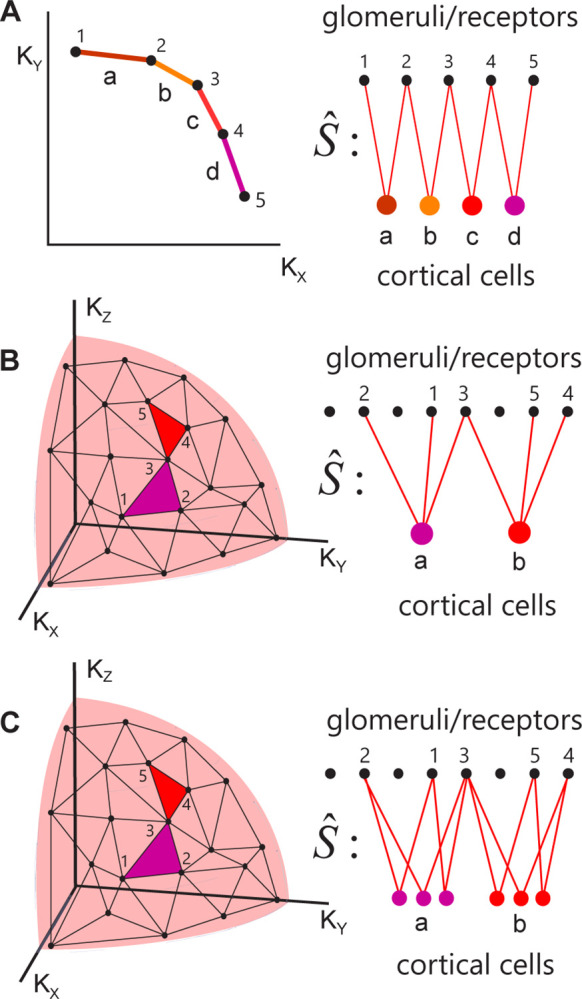
Suggested feedforward circuit which can process the primacy information. **A**. *Left*: Primacy hull for *D* = 2 and *p* = 2; a, b, c, d are discriminable odor identities. *Right*: connectivity between ORs 1, 2, …5 and cortical cells (insect mushroom body cells) corresponding to different odor identities. The glomeruli from the same primacy sets converge to the same cortical cells. **B.** The same for *D* = 3 and *p* = 3. Only two example simplexes are shown. **C.** Subprime connectivity model. Individual cortical cells represent the faces of primacy simplexes (sides of the triangles). Individual odor identities are encoded by populations of neurons marked ‘a’ and ‘b’.

The coactivation of primary ORs could be detected by feedforward connectivity from ORs/glomeruli to higher processing centers, if the connectivity contains information about the primacy hull. In this mechanism, the projections from individual primacy sets of ORs/glomeruli would converge on distinct cells in the piriform cortex (PC) or the mushroom body (MB) in insects (Fig 3). PC/MB neurons are expected to respond when the corresponding primacy ORs are co-activated (Fig 3A and 3B). This prediction suggests that, the feedforward connectivity contains high-order correlations induced by the presence of the primacy hull in OR affinities. It also suggests that the connectivity is related to the OR responses: PC/MB cells may integrate inputs from the OB/AL neurons with the strongest affinity for an odorant. Below, we will test this prediction using recent AL-to-MB connectivity and OR-odorant affinity data from *D*. *Melanogaster*.

In the connectivity model proposed above (Fig 3A and 3B), only a single PC neuron, corresponding to a primacy simplex, responds to the presentation of an odorant. Experimental data in mice shows that the PC contains about *N*_*PC*_≈2·10^5^ pyramidal neurons out of which about 5%, i.e. *n*_*a*_~10^4^, respond to an odorant [39, 40]. One way in which a primacy model can generate 10^4^ responses is if individual neurons in the PC represent faces of the primacy simplex rather than the full *p*−1-simplex itself (Fig 3C). These faces are also simplexes themselves. For example, a triangle or a 2-simplex contains three sides as faces, which can be viewed as 1-simplexes. A tetrahedron, a 3-simplex, contains four triangles (2-simplexes) as faces (Fig 2A), etc. Overall, the number of *n* -point faces of a primacy (*p*−1) -simplex is given by the binomial equation

$$F_{n,p}=\frac{p!}{n!(p-n)!}$$ (6)

For example, a 4-vertex simplex (tetrahedron) contains 4!/3!/(4–3)! = 4 3-vertex faces (triangles) and 4!/1!/(4–1)! = 6 2-vertex faces (segments, Fig 2A).

We propose that the responses of cells in higher brain regions such as PC or MB reflect the activations of simplexes that are subsets or faces of the primary simplex corresponding to the presented odorant identity. This proposal may explain the large population of cells activated in the PC and MB in response to an odor. For example, in the mouse PC, an odor activates *n*_*a*_~10^4^ cells [39, 40], which roughly corresponds to the primacy number of *p* = 16 and the number of converging connections onto a PC cell *n* = 8: *F*_8,16_≈1.3·10^4^. Below, we will refer to the faces of the primary simplex as the *subprime* simplexes. The complete set of subprime simplexes of a given degree uniquely represents the primacy simplex. For example, in Fig 2A, the set of four triangles uniquely represents the primacy tetrahedron. Such a coding scheme may provide robustness to noise. A distributed representation of an odor is much less sensitive to the failures of individual neurons to be activated. If a single neuron in the PC or MB represents an odorant identity (Fig 3A and 3B), silencing this neuron will eliminate the perception of this smell. In the case where individual neurons represent the subprime simplexes corresponding to the same odor (Fig 3C), a failure of activation of individual neurons due to the presence of noise can be compensated by a pattern completion mechanism implemented by associative circuits in the PC [41]. This can be accomplished if the subprime simplexes corresponding to the same primacy simplex are connected by synapses with positive strength, similarly to a Hopfield network. Overall, we suggest that cells activated in the PC represent the faces of the primacy simplex corresponding to the stimulus identity. These representations can be generated by the feedforward OB-PC (or AL-MB in insects) connectivity that contains the primacy hull structure in the weight matrix and may be facilitated by the recurrent connectivity in the PC.

### Experimental predictions

According to our model, the AL-MB or OB-PC connectivity is expected to contain a distributed representation of the primacy hull. Specifically, we expect i) connectivity data to have a low-dimensional component that is consistent across members of the same species and ii) individual MB/PC neurons to integrate inputs from high affinity ORs to an odorant (primacy sets).

Below we test these two predictions in the fruit fly (*D*. *Melanogaster*) using two independent connectivity datasets from two individual flies and an OR-odorant affinity dataset. We will use the terms OR and glomerulus interchangeably to refer to a genetically defined olfactory information channel consisting of a single glomerulus and its homotypic OR.

### Using existing data on connectivity and OR affinities to test the primacy model

**Fig 4:**
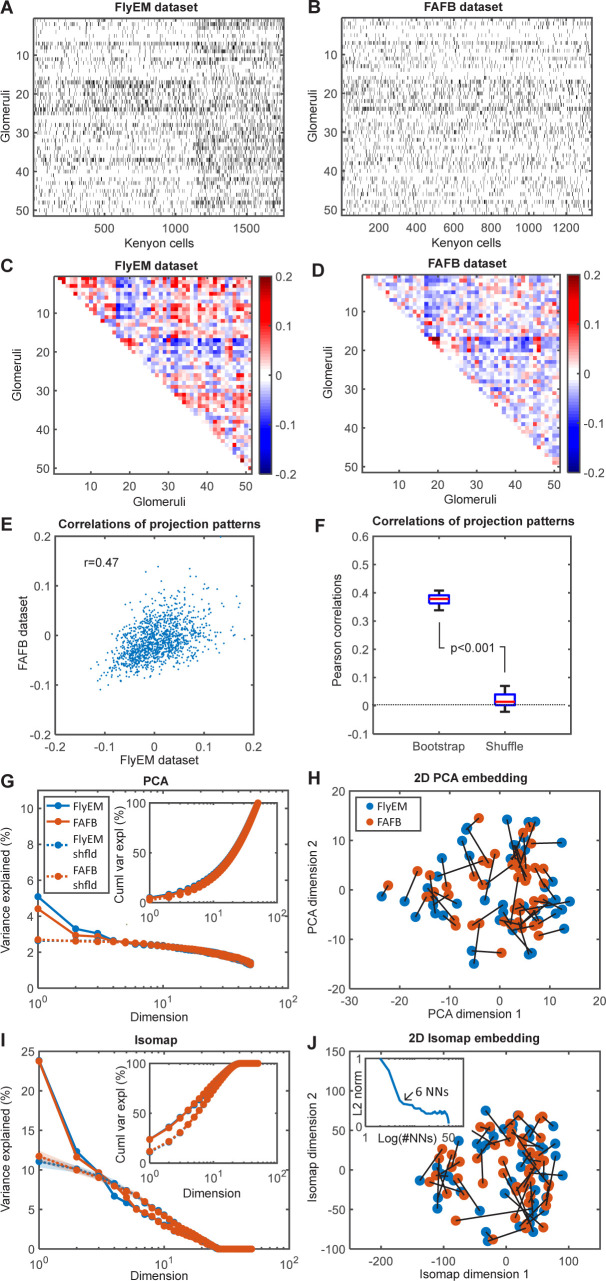
Non-random low-dimensional structure in PN-KC connectivity that is conserved across animals. **A**, **B**. Glomerulus-KC connectivity matrices from FlyEM and FAFB datasets. **C, D**: Glomerulus-glomerulus connectivity similarities (Pearson correlations of connectivities). **E**. Glomerulus-glomerulus connectivity similarities in two datasets against each other. The correlation in glomerulus-glomerulus connectivity similarities is r = 0.47 (p<0.01). **F**. Similarity between datasets disappears if one of the datasets is shuffled while preserving the connectivity matrix in- and out- degrees (right)[44]. We observed that the average correlations for bootstrapped connectivity data in which KCs were selected with repetitions is somewhat lower than for the intact data in panel E. **G**. Variance explained per dimension as a function of the PCA dimension (inset–total cumulative variance explained). PCA analysis shows that the first two linear dimensions are significantly different from random. **H**. Connectivity matrices in two datasets projected onto the first two dimensions. Points represent individual glomeruli. The same glomeruli in two datasets (different animals) are connected by black segments and reside near each other in the 2D embedding suggesting that the first two dimensions of the connectivity matrix are conserved across datasets. **I, J**. The same analysis using a non-linear low-dimensional embedding technique (Isomap) shows that the first two dimensions in the connectivity data are both different from random (**I**), explain more variance in the data than the linear algorithm (PCA), and are conserved across datasets (**J**). The number of nearest neighbors for the Isomap algorithm (inset) was chosen as described in Methods H in S1 Text.

In the fly, OR activity is relayed to the MB via projection neurons (PNs); a majority of PNs receive their inputs from only a single glomerular channel. Each OR/glomerular channel is associated with several PNs. The principal cells of the MB, the Kenyon cells (KCs), are the major targets of PN axons, with a single KC integrating ~5–6 glomerular/OR type inputs. Two recent studies describe PN-KC connectivity in two adult flies. The FlyEM dataset contains the PNs originating from all 51 olfactory glomeruli and terminating at a large number of KCs (N_KC_ = 1784 out of a total estimated ~2000–2500 KCs per MB hemisphere) [42]. The FAFB dataset includes connectivity for 51 glomeruli and 1344 KCs [43]. Using this binarized connectivity data (Fig 4A and 4B), we can interrogate the logic governing KC integration of OR channels.

To test the prediction that the PN-KC data contains a low-dimensional component that is consistent across individual flies (experimental prediction i), we first compare the FlyEM and FAFB connectivity matrices. We aligned the two data matrices along the PN dimension by grouping the PNs based on the identity of the glomeruli/ORs that they receive the primary input from. As there is no simple way to align KC identities across different animals, we started with analyzing the *similarity* of connectivity between individual glomeruli. These similarities were defined as the Pearson correlation coefficients between pairs of glomeruli in terms of their projections to KCs computed for both FlyEM and FAFB datasets (Fig 4C and 4D). We observed a substantial correlation between the similarity matrices for the two datasets (R = 0.47, Fig 4E). The correlation was eliminated by shuffling either of the connectivity matrices (Fig 4F). Following Ref. [44], we used a shuffling procedure that preserves the number of connections from each glomerulus to all KCs and from each KC to all glomeruli (KC in- and glomerulus out-degrees). The presence of a significant correlation in connectivity between the two animals suggests that the glomerulus-KC connectivity matrices share a common structure between the two individual animals.

Above we hypothesized that if ORs sample a low-dimensional subspace of the affinity space, this subspace should be reflected in the connectivity structure consistent across members of the same species (experimental prediction i). To further characterize this common structure in the two connectivity datasets, we applied linear and nonlinear dimensionality reduction techniques to the glomerulus-glomerulus similarity matrices. Linear dimensionality reduction methods, such as the principal component analysis (PCA), place the objects (here–glomeruli/ORs) on a flat surface, while nonlinear methods, such as Isomap, arrange them on a curved manifold of a given dimension. The quality of these embeddings can be assessed by the variance in the data explained by the embeddings. We applied both methods to the glomerulus-glomerulus connectivity similarity matrices (Fig 4C and 4D). First, we applied the PCA (flat) method (Fig 4G and 4H). We found that the first two dimensions of the PCA space of the data explain more variance than those of the shuffled datasets (again, shuffling was performed in a way that preserves the in- and out-degrees for each KCs and PNs respectively, as in Ref. [44]) (Fig 4G). We also found that the same glomeruli/ORs in the two flies resided near each other when placed in the first two dimensions of the PCA space (Fig 4H, root mean squared deviation (RMSD) of the 2D positions between the two datasets is 5.9, versus RMSD = 13.5 for the randomly shuffled data, p<10^−6^, t-test). Our further analysis indicates that the first dimension in the embedding space is correlated with the sensitivity of olfactory receptors for food, while the second dimension has no clear functional significance (S1 Fig). Both the first and the second dimensions are not obviously related to the degree of connectivity of the KCs and instead are produced by the glomeruli projecting to specific subsets of KCs (S2 Fig).

The first two *flat* dimensions of the data explained only about 9% of the variance in the connectivity data. As suggested by earlier work on the embedding of olfactory spaces [8, 9], the OR affinities may be better approximated by a curved low-dimensional manifold. To account for this possibility, we used the Isomap algorithm [45] (see Methods H in S1 Text). The first two dimensions of the curved Isomap space account for about 39% (FlyEM) or 35% (FAFB) of the variance in the connectivity data (Fig 4I, as quantified by the variance explained within the Isomap manifold). The datasets contain more variance along the first two dimensions (Fig 4I) as compared to the shuffled data with preserved in- and out- degrees [44]. In addition, the individual glomerular connectivities, when embedded into a curved 2D manifold are situated in closely matching locations in the two flies (Fig 4J, RMSD = 47.2 versus RMSD = 83.5 for shuffled data, p<10^−5^, t-test), suggesting that the structure of PN-KC connectivity is preserved between different individual animals within the same species. Overall, our findings demonstrate that the glomerulus-KC connectivity contains low-dimensional structure, as hypothesized in our primacy theory. Such connectivity structure can be identified in two different animals, suggesting that it is specified genetically. The structure is eliminated by the random shuffling of data which argues against the hypothesis of fully random OR-KC connectivity.

**Fig 5:**
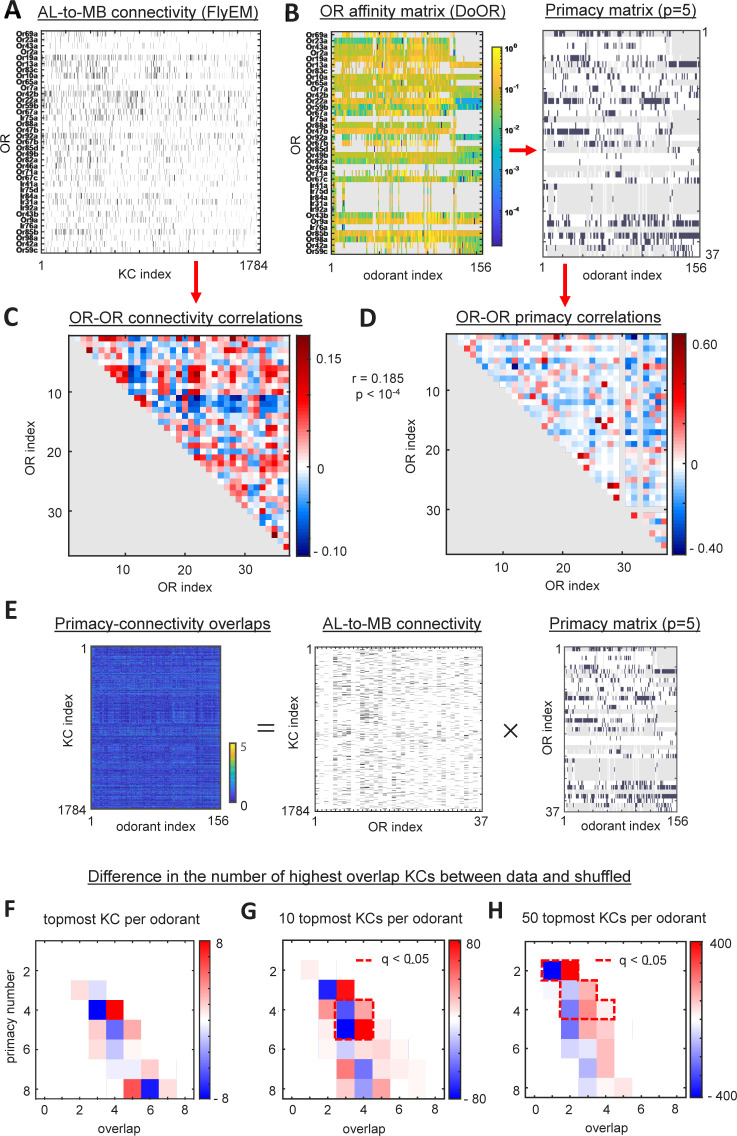
Comparing connectivity and affinity data. **A**. OR-KC connectivity matrix (FlyEM dataset). **B**. *Left*: OR-odorant affinities (from DoORv2 dataset). Gray represents missing values of affinities. *Right*: OR-odorant primacy matrix for p = 5. **C**. OR-OR similarities (Pearson correlations) computed from connectivity data for 37 ORs represented in affinity data. **D**. OR-OR similarities (Pearson) computed from affinity data. The off-diagonal elements of matrices in **C** and **D** are correlated (R = 0.185, p < 10^−4^). **E**. Schematic showing the overlap matrix computed by matrix multiplication of connectivity and primacy matrices (p = 5). **F**. The difference in the number of actual overlaps of a given degree and the number of overlaps for a randomly shuffled connectivity matrix [44]. The difference is computed for different primacy numbers. Only the KCs with the highest overlap per odor (gKC) are considered. No statistically significant difference is observed. **G**. As in (**F**), but for the 10 topmost KCs per odor. Statistically significant differences are enclosed by a dashed red line (FDR<0.05). **H**. As in (G), but for the 50 topmost KCs per odor.

Is this conserved structure related to OR affinities for odorants? Above, we suggested that individual KCs integrate inputs from ORs displaying high affinities for certain odorants (Fig 3). This hypothesis, listed above as prediction ii), can be tested by comparing the connectivity data (FlyEM, Fig 5A) with the DoOR dataset[46] (Fig 5B). DoOR contains the most substantial description of Drosophila OR-ligand affinities available. Although not all OR-ligand pairs are present in the data (Fig 5B), we can compute approximations of the primacy sets for 156 odorants. To compare the odor response data to the connectivity data, we first performed an analysis of the OR-OR similarity matrices in terms of their connections to KCs and in terms of their participation in the primacy sets. If KCs integrate inputs from high-affinity (primacy) ORs, the matrix of OR-OR similarities in connectivity (Fig 5C) is expected to be correlated with the OR-OR primacy similarities (Fig 5D). First, from the OR affinity matrix (Fig 5B, left panel), we computed the primacy matrix for primacy number p = 5 (Fig 5B right panel). In this matrix, each element is equal to one if the given OR belongs to the set of p = 5 strongest responders to the given odorant and zero otherwise. Using this primacy matrix we calculated the OR-OR correlation matrix and compared it to the OR-OR connectivity correlation matrix for the subset of glomeruli with a single OR input. We found that the two matrices are significantly correlated (R = 0.185, p<10^−4^), which suggests a relationship between the high-affinity sets of ORs and the OR-KC connectivity. The low value of the Pearson correlation is expected here since we do not have access to the entire set of odorants ethologically relevant to the species and not all OR-ligand pairs are present in the data (Fig 5B). The results of this analysis carried out for other values of the primacy number p = 1–8 are shown in S5 Fig.

Can we directly compare the OR-KC connectivity matrices to the primacy sets of the odorants present in the DoOR dataset? To perform this analysis, for each KC, we computed the overlaps (matrix product along the shared dimension) between the connectivity data and the primacy sets of ORs for each of the 156 odors present in the DoOR data. We can then find, for every odorant, a ‘grandmother’ KC (gKC, Fig 3A and 3B), i.e. the KC for which the overlap between its connectivity with the primacy OR set for this odorant is the highest. Using this procedure, we can find 156 gKCs for each of the odorants present in the DoOR dataset and the 156 corresponding overlaps. According to primacy theory, the KCs should integrate inputs from primacy sets, so the overlaps between the gKC connections and the primacy sets are expected to be higher than for random connectivity. We find, however, *no* enrichment in the overlaps between connections and primacy sets for 156 gKCs identified in data compared to a randomly shuffled connectivity [44] (Fig 5F, FDR adjusted p-value (q-value) > 0.05). This finding suggests that the processing of the primacy information in the AL-MB network may not be based on the ‘grandmother’ KC mechanism (Fig 3A and 3B).

Individual odorants activate >50 KCs in the MB [47, 48], suggesting that the population of KCs may represent different subsets of the primacy set for the odorants (Fig 3C). We suggested that these subsets can be viewed as individual faces of the primacy simplex [Eq (6), Fig 3C]. This mechanism is more robust than the one based on gKCs, in which only one cell is activated in the MB representing the primacy simplex. To test this population-based mechanism, for each odorant, we identified not one but 50 KCs with the highest overlaps between connections and primacy sets of ORs. We thus evaluated 156 x 50 values of overlaps between connectivity and primacy sets for the odorants in the DoOR dataset. We have found a substantial enrichment in the number of higher overlap scores for the FlyEM connectivity compared to randomized connectivity matrices [44] (Fig 5H, FDR q<0.05). The enrichment can be seen in the presence of the red (positive) band to the right of the blue band in Fig 5H indicating a larger number of higher overlaps compared to the random case. The enrichment in the overlaps between OR-KC connectivity and primacy sets is observed for responsive KC population sizes as low as 10 (Fig 5G) and primacy numbers in the range between 2 and 5. These findings indicate that, for individual odorants, a population of KCs has connectivity that is correlated with the primacy sets of ORs for this odor (Fig 3C), rather than individual gKC (Fig 3A and 3B). This correlation is significantly higher than that observed for randomized connectivity with preserved in- and out-degrees. We analyzed the sensitivity of our analysis to the missing entries in the DoOR dataset in S6 Fig. Collectively, our analyses support our hypotheses regarding the processing of primacy information in the fly olfactory system by confirming i) the presence of a low-dimensional structure in the feedforward connectivity that is shared across individual members of a species, and ii) that individual KCs integrate inputs from ORs with high affinity for an odorant (elements of primacy sets).

## Discussion

How can the nervous system link the representation of the same odorant at low and high concentrations? According to the primacy model, the odor identity is encoded by the OR types with the highest affinity for a given odorant, i.e., the primacy set of ORs. In air-breathing animals, odor exposure is defined by a sniff cycle, and the primacy set is activated at the beginning of a sniff cycle. As such, the primacy set is expected to be invariant to the ambient odor concentration and can link odor identity percepts across concentrations.

Hopfield proposed a model that attributes an odor identity to the sequence of receptor neuron activation [49]. In his model, an increase in odorant concentration leads to a temporal shift in the entire OR activity pattern [30, 50–52]. However, it is not clear how such a model can process the additional signals from the receptors that were not activated at low concentrations but are recruited at higher concentrations. Alternative models of concentration invariant identity assignment based on the normalization of bulbar responses may only partially solve this problem [53–56]. Indeed, such a normalization requires integration of the inputs across all channels, or glomeruli [57] including those that are activated later in the sniff cycle [58]. Such mechanisms seem to preclude odor-guided decisions based on early olfactory inputs [25, 59].

Several lines of experimental evidence support the primacy coding mechanism. To discriminate two odorants, animals use the information accumulated during a short temporal window (~100 ms) at the beginning of the sniff cycle [25] and make these decisions before the entire ensemble of olfactory glomeruli is activated [58]. These odorants were presented at random concentrations. Similarly, the concentration-invariant cortical representations are found to be formed early in the sniff cycle [27]. A recent study, in which the timing of receptor activation was controlled optogenetically, demonstrated higher relevance of early activated glomeruli for sensory object identification [28]. Earlier studies in insects showed that the neural activity trajectories, in response to odor stimuli, diverge quickly for different odorants, but, initially, go together for the same odorant at different concentrations [60]. These results are consistent with the primacy coding mechanism.

The primacy model defines the primacy set as either the set of the most sensitive receptor types (affinity primacy) or as a set of the earliest activated receptor types/glomeruli (temporal primacy). In this study, we assumed that affinity and latency are highly correlated. While this is a reasonable assumption, OR activation latency may be affected by factors other than affinity, such as the solubility of an odorant in the mucosal layer [61] or the receptor distribution in the epithelium. Although the conclusions of the primacy theory are valid for both affinity and latency-based coding mechanisms, different forms of primacy may exist in different species. For example, in animals with a slow sniffing cycle, such as fish, affinity-based primacy may be a viable mechanism.

In addition to the *primacy coding hypothesis*, we proposed the hypothesis that the olfactory system samples a low-dimensional subspace in the space of odorant properties. We concluded that if olfactory space is low-dimensional and odorant identities are encoded according to a primacy code, evolutionary dynamics will drive ORs to reside along a thin high-affinity boundary that we call the primacy hull. In this model, the ORs that do not have a high affinity for any of the features of interest to the olfactory system will ultimately be pseudogenized. Thus, both the primacy and low-dimensionality assumptions are necessary for the primacy hull to exist. If, for example, the olfactory space is instead high-dimensional, the primacy mechanism may still be valid [25]. In this case, every OR can be a member of the primacy set, and, thus, evolution will favor the retention of all of the ORs in the genome. The hypothesis of the low-dimensional stimulus space may also be compatible with alternatives to the primacy coding mechanisms. Primacy and low dimensionality are therefore two independent assumptions of our model.

Besides these two main assumptions, we have made many other simplifications that are frequently made in the olfactory literature. For example, we assumed that receptor activation by odorant mixtures follows a simple linear-nonlinear (LN) relationship [Eq (2)]. Although this approximation is conventional [36, 62, 63], in light of the significant recent work exploring the effects of non-linear interactions between mixture components [29, 30, 64–66], we present an analysis of a more complex mixture model in Methods D in S1 Text. In the low concentration regime, in which the primacy sets are determined, we recover OR activations similar to Eq (2), which justifies our use of this equation to describe primacy. The formation of the primacy hull is based on the assumption that most of the ORs that do not carry an olfactory function are eliminated in the course of evolution (pseudogenized). This assumption seems to be supported by the almost complete loss of ORs by aquatic mammals, such as dolphins and toothed whales [67]. If a substantial fraction of ORs were involved in non-olfactory functions, such a loss would not be possible.

The early identification of a predator or food source is a factor of evolutionary importance; by relying only on the highest affinity or shortest latency OR channels, the primacy code optimizes for the speed of percept formation. On the timescales of a single sniff, the primacy coding provides a quick and robust mechanism for identifying an odorant irrespective of the concentration at which it is encountered. An air-breathing animal may also increase the accuracy of its odor identification by sampling over several sniff cycles and integrating the information to support a slower but potentially more accurate olfactory decisions. This speed-accuracy tradeoff in olfaction has been previously explored [68] and does not contradict the primacy mechanism, which operates on the time scales of a single sniff.

The primacy code involves the selective integration of early (primary) OR responses using structured feedforward circuit mechanism (Fig 3). After the first primacy set of ORs is activated, the effects of subsequent primacy sets need to be eliminated, for example, through the use of recurrent inhibition either in the OB [26] or in the cortex [25, 27]. In the OB, the early activated glomeruli send signals to the cortex via mitral/tufted (MT) cells. Early MT cells activate an inhibitory bulbar network and may scramble or suppress the information from later activated MT cells [26]. Further in the cortex, early responses drive activity in a population of excitatory cells, which activate inhibitory interneurons via recurrent connections [27]. This results in ‘global’ inhibition in the PC, which suppresses the contributions from later responding (non-primary) ORs. This mechanism may implement a p-winner-takes-all (pWTA) circuit [25]. The primacy number could therefore reflect the average number of OR inputs necessary to drive global inhibition and produce a stable representation in the PC or MB. The primacy number may not be fixed across different odorants. The number of OSNs and MT cells per OR type can vary widely [69, 70] leading to differences in the excitatory drive provided by different OR channels. Assuming a fixed threshold for activating the global inhibitory shutdown of later responses, this would imply that the p-number for an odorant depends on the OR channels that it activates early in the sniff cycle. For example, if an odorant primarily activates ORs that are overrepresented at the OSN, glomerular, and MT levels at the start of the sniff cycle, one expects the primacy number to be lower than the average. The effective primacy number may also change if the cortical network structure results in different effective thresholds for different odorants or via adaptation of the number of OSNs per OR channel at the epithelium to sensory scene statistics [69]. In such cases, a stable odorant representation in the PC/MB may still be achieved by pattern completion networks [32, 71], which can compensate for degraded or incomplete inputs.

Our model makes two specific predictions regarding the structure of the connectivity in the early olfactory system (AL to MB in insects or OB to PC cells in vertebrates). We proposed that primacy information is processed by neurons in the target region (MB or PC) integrating inputs from ORs belonging to primacy sets. In this model, target neurons respond to activations of subsets of the primacy set (Fig 3C), which makes the representations of odor identity in the target region robust to noise. Indeed, if a PC neuron corresponding to a particular subset of the primacy OR set is not activated due to fluctuations in the inputs, this PC neuron may still be pushed over the activation threshold by the associative excitatory circuit in the PC. This prediction yields at least two corollaries. First, the low-dimensional structure of the odor space tessellated by the primacy sets should be present in the connectivity structure. As such, it can be revealed by a conventional dimensionality reduction method, such as PCA or Isomap. Second, the feedforward connectivity should be correlated with OR responses: PC/MB neurons should tend to receive inputs from subsets of the primacy sets for specific odorants. We have tested these predictions using two recently obtained datasets on connectivity in the *D*. *Melanogaster* olfactory system [42, 43] and the data on OR-odor affinities [46]. First, we found that connectivity data from two individual flies contains a similar low-dimensional structure. Second, we found that MB Kenyon cells are more likely to receive connections from high-affinity (primacy) sets of receptors for individual odorants. These observations are consistent with the primacy coding hypothesis.

Several theories have been proposed to describe the connectivity in the *D*. *Melanogaster* olfactory system. Many models assume that the early olfactory system performs a form of compressed sensing (CS) [33, 72–77]. These models assume that odorant mixtures can be viewed as sparse signals in terms of their molecular composition. These signals are compressed into a denser vector of OR responses which is accomplished by multiplying them with the ligand-OR affinity sensing matrix [Eq (2)]. Due to the requirement of the CS algorithm, the affinity matrix is expected to be random, or, at least, lack low-dimensional structure [78]. This approach allowed us to estimate the number of OR needed for lossless sparse encoding using the Donoho-Tanner theorem [33, 78]. In many of these models, the function of the early olfactory system is to perform the decoding of the compressed signal. To accomplish this step, in these models, the feedforward OB-PC (or AL-MB) connectivity represents the sensing matrix, i.e. the odorant-OR affinity matrix. Since the CS approach requires the sensing matrix to be random, these theories involve unstructured connectivity between OB/AL and PC/MB. Zavatone-Veth et al. [72] have used neural recordings to constrain the variance of the elements of the sensing matrix. The low-dimensional structure of the affinity between ligands and receptors was not incorporated into that model. Zhang and Sharpee [76] proposed a feedforward-only model for decoding the sparse vector of molecular concentrations. As in other CS-based theories, the feedforward connectivity in this model mimics the random affinity matrix. A straightforward prediction of the CS-based models would include a correlation between connectivity and affinity matrices (Fig 5C and 5D), with a lack of low-dimensional structure in connectivity (Fig 4I). Grabska-Barwińska et al. [37] proposed a theory in which PC cell activity represents marginal probabilities of single molecules to be present in the mixture. The OB-PC connectivity in their model is given by the OR-ligand affinity matrix. Pehlevan et al. [79] developed a theory in which the AL-MB network implements an unsupervised clustering algorithm which is derived from the k-means cost function. The vector of PN-KC synaptic weights converging onto a given KC represents the corresponding cluster centroid. The implications of their learning rule for the feedforward connectivity are yet to be explored. Several groups have proposed that the fruit fly olfactory circuit solves the similarity search problem using a variant of the locality-sensitive hashing algorithm [47, 80]. This algorithm relies on a random and sparse AL-MB matrix. Thus, a significant class of theories explores the implications of unstructured (random) connectivity for olfactory processing [25, 81–83]. The interpretation of the fruit fly connectivity data in the context of the theories based on random sensing deserves further thorough investigation. Kepple et al. [33] have used duality to derive the circuit implementing CS using primacy conditions. They found that the feedforward connectivity matrix should contain primacy sets. In their case, the affinity matrix was unstructured and of a high rank as in the other theoretical studies. Here, we generalized this idea to the feedforward connectivity in the case of low rank affinity matrix (Fig 3). This prediction was supported by the fly connectivity data (Figs 4 and 5). The optimal algorithm for deducing the relevant features of the olfactory stimuli in the case of low rank affinities is beyond the scope of the present study.

Recently, Zheng et al. [43] characterized the structured component in the PN-to-MB connectivity matrix. They found a structure derived from a set of food-related glomeruli converging on KCs more frequently than would be expected under null models. This result is in agreement with the analysis presented here. First, this over-convergence is reflected in the principal component analysis of the AL-MB connectivity matrix, in which food-related glomeruli are colocalized along the first dimension (S1 Fig). The second principal component of connectivity, however, is not related to food (S1 Fig). As both the first and the second principal components are statistically significantly different from random, the over-convergence of food-related glomeruli does not fully account for the observed structure in the connectivity matrix. Second, food-related glomeruli may have a wider role in the combinatorial coding of other not necessarily food-related odors. We may interpret these over-converging glomeruli as the important vertices/simplexes of the primacy hull encoded in the connectivity.

Overall, we have explored the implications of the primacy model [25] which yields concentration-invariant odor identity representations based on the ORs most sensitive to a given odorant. We assumed that the receptors sample a low-dimensional space of odorant properties relevant to the organism’s fitness. We argue that the evolutionary pressure to represent odorants according to a primacy code leads to the arrangement of OR types along a high-affinity surface called a primacy hull. Our multimodal analysis of fly olfactory datasets supports our predictions about the implications of primacy coding for olfactory circuit organization.

## Methods

Our methods are described in S1 Text.

## Supporting information

S1 Text Supplementary methods. (DOCX)

S1 Fig Functional significance of non-random features of connectivity in FlyEM and FAFB datasets. (A) Glomeruli placed in the connectivity’s 2D PCA space. The same glomeruli in two datasets are connected by lines. Glomeruli are colored according to their function as indicated. The first PC of connectivity appears to be related to food-sensitive glomeruli, while the second PC is unrelated to food. (B-D) Three first PCs for the two datasets plotted against each other. The first two PCs (B and C) are conserved between FlyEM and FAFB datasets (R = 0.92 and 0.78), while the third PC appears to be random. This indicates that only the first two PCs of connectivity are conserved across individual animals. (E-H) For randomly shuffled connectivity matrices, none of the principal components are conserved across individuals.
(PDF)

S2 Fig The first two PCs of connectivity cannot be explained by the in-degree of the KCs. Instead, they are related to the connectivity structure. (A) Binarized connectivity matrix in FlyEM dataset with both ORs and KCs sorted according to their contribution to the first PC (PC1). ORs with similar PC1 appear to have stronger connectivity, suggesting that the structure of OR-KC connections determines the contribution of ORs to a PC. (B) The number of connections made by KC in the binarized matrix does not have a clear monotonic dependence on PC1. Thus, the first PC is not produced by differences in the KC in-degree. (C, D) Same for PC2. A diagonal band along the diagonal in the sorted connectivity matrices in (A) and (C) indicates that ORs are connected to specific groups of KC, which determines both PC1s. Thus PC1 and PC2 emerge from a specific connectivity structure. (E-H) The same analysis in the randomized matrices shows that the first PC is correlated with the KC in-degree (F).
(PDF)

S3 Fig Results of brute force alignment of connectivity datasets. (A) Two connectivity datasets aligned using the simulated annealing algorithm. (B) Same for two randomly shuffled connectivity matrices. (C) Even randomly shuffled connectivity matrices share ~77% of synapses, when aligned. Unshuffled connectivity matrices share ~78% synapses. (D) Hamming distances between aligned KCs. Only 16 KCs are an exact match (H = 0) versus 6 KCs in the randomly shuffled case.
(PDF)

S4 Fig Glomeruli and their cognate odorant receptors. Each glomerulus in the *Drosophila* antennal lobe receives input from specific OR types. The majority receive input from only a single odorant-responsive receptor type, however some glomeruli, e.g. DL1, receive converging input from >1 OR type. *Left*: OR types present in the DoOR dataset are represented by a filled green circle, while a blue circle represents those ORs missing from the DoOR. *Right*: Glomeruli that are included in Figs 4 and S1 are labeled in bold and those that are included in our analysis of DoOR data (Fig 5) are labeled in bold red. Based on data from [13] (S1 Text).
(PDF)

S5 Fig Sensitivity of correlations (Fi 5C and D) to the choice of the primacy number. *Left*: OR-OR Pearson correlation matrices for FlyEM and FAFB connectivity data (37 glomeruli). *Right*: OR-OR Pearson correlation matrices for primacy sets in DoOR affinity data shown for a range of primacy numbers. Correlations between connectivity and primacy sets are reported for FlyEM (in purple) and FAFB (in green). Statistically significant correlation coefficients are recorded for a range of primacy numbers.
(PDF)

S6 Fig Sensitivity of our findings in Fig 5 to missing data. We first generate i) a surrogate affinity matrix with similar statistical properties to the DoOR dataset and ii) a related surrogate connectivity dataset. Both datasets are related via the same primacy hull (see Section S10 above). We impose the same missing structure on the surrogate affinity data as observed in the empirical DoOR data and observe that the proposed overlap test can indeed detect the shared primacy hull.
(PDF)
