# Supplementary material for: The primacy model and the structure of olfactory space
Source: PLoS Comput Biol. 2024 Sep 10;20(9):e1012379. doi: 10.1371/journal.pcbi.1012379 (PMC11423968; doi:10.1371/journal.pcbi.1012379)
Supplement: S1 Text — (DOCX) [file pcbi.1012379.s001.docx]

**Supplementary Methods**

**Methods A. Generating primacy hull in $D$ dimensions** $P_{D,p}$**:** A large number of points $\left\{\overrightarrow{r}_{i}\right\}$ representing ORs are distributed in the positive orthant in $D$ dimensional space. From these points, the points are selected via the following algorithm: i) generate a random unit vector $\overrightarrow{q}$ in the positive orthant, ii) compute $\overrightarrow{q}\overrightarrow{\cdot r}_{i}$ for all points indexed by $i$ iii) select the $p$ points with the highest scalar product (the simplex) and add them to a list, $L$. Repeat steps (i)-(iii) until $L$ does not change. The resulting $N$ points (OR types) are the vertices of the primacy hull $P_{D,p}$.

**Methods B. Generating simulated affinity data** $K$ **containing** $P_{N,D,p}$**:** Given a primacy hull $P_{N,D,p}$, vectors representing ORs can be collected into a matrix $R^{N\times D}$**.** Similarly, a panel of odorants represented by random unit vectors (always in the positive orthant) $\left\{\vec{q}_{j}\right\}$ where $j=1,...,N_{odorants}$ can be collected as rows of the matrix $P^{D\times N_{odorants}}$. Affinity data of dimensions $N_{odorants}\times N$ is simulated as $K=RP$ (equation 4)**.**

**Methods C. Linear model for OR responses to a mixture of odorants:**

Consider the mass-action law for an OR binding odorant number $o$:

$$dR_{ri}/dt=-\gamma_{i}R_{ri}+\alpha_{ri}C_{i}R_{r}$$

Here $\alpha_{ri}$ and $\gamma_{i}$ are binding and unbinding rates for receptor $r$ and ligand $i$, and the number of unbound (available) receptors of type $r$ is given by

$$R_{r}=R_{r0}-\sum_{i} R_{ri}$$

Here $R_{r0}$ is the total number of OR molecules of type $r$ exposed to odorant binding. Because in the equilibrium $dR_{ri}/dt$ and $R_{ri}=\alpha_{ri}c_{i}R_{r}/\gamma_{i}=K_{ri}c_{i}R_{r}$, the total number of unbound receptors can be found from equation

$$R_{r}=R_{r0}-R_{r}\sum_{i} K_{ri}c_{i}$$

We thus obtain $R_{r}=R_{r0}/(1+\sum_{i} K_{ri}c_{i})$ and the number of activated receptors

$$\sum_{i} R_{ri}=R_{r0}-R_{r}=\frac{\sum_{i} K_{ri}c_{i}}{1+\sum_{i} K_{ri}c_{i}}$$

By assuming that the activity of the cell reflects the OR activation we obtain

$$\frac{f_{r}}{1-f_{r}}=\frac{\sum_{i} R_{ri}}{R_{r0}-\sum_{i} R_{ri}}=\sum_{i} K_{ri}c_{i}$$

i.e. equations (1)-(2) of the main text.

**Methods D. Non-linear OR responses to mixtures and implications for the geometry of the primacy hull:**

In the main text, we assumed that an OR is activated if the following holds for its responses to a vector of concentrations $c_{j}=cq_{j}$ (here $\left\|\overrightarrow{q}\right\|=1$):

$$\frac{f_{i}(c)}{f_{\max}^{i}}\approx c\sum_{j} K_{ij}q_{j}=\theta_{i}$$

Here $\theta_{i}\ll1$ is the threshold, for OR number $i$, at which this activation becomes noticeable to the system, and $c$ is the overall concentration of the mixture. When applied to every OR, Equation allows us to determine both the set of primary receptors and the concentration of the mixture at which this set is fully activated. Here, we will identify the limits of applicability of this equation if the OR activation is determined by a more complex function that models mixture interactions.

A number or studies have shown that OR responses to mixtures of odorants are not always well captured by the linear-non-linear model used in the presentation of the primacy hull in the main text [[1-5](#_ENREF_1)]. More complex models of OR-ligand dose-response properties account for non-linearities in OR responses induced by mixture interactions, such as antagonism, synergy, and masking [[2](#_ENREF_2), [4](#_ENREF_4)]. These models typically contain an additional parameter - $\eta_{ij}$ - which describes the *efficacy* with which a ligand $j$ activates OR $i$. The general form for the activation of OR $i$ by a mixture containing $M$ components (obtained by integration of the rate equations from a two-step reaction model), is given by:

$$f_{i}(c)=\frac{f_{\max}^{i}}{1+\left(\frac{1+c\sum_{j=1}^{M} \kappa_{ij}^{-1}q_{j}}{c\sum_{j=1}^{M} \eta_{ij}\kappa_{ij}^{-1}q_{j}}\right)^{n}}$$

where the concentration of component $j$ is given by $c_{j}=cq_{j}$, $j$ runs from 1 to $M$, the number of monomolecular ligands in the environment and $q_{j}$ is the partial concentration of $j$ in the mixture, $n$ is the Hill coefficient and $\kappa_{ij}$ is the affinity of ligand $j$ for OR $i$. This function can be approximated (for low concentrations of odorant) as:

$$\frac{f_{i}(c)}{f_{\max}^{i}}=\frac{c\sum_{j=1}^{M} \eta_{ij}\kappa_{ij}^{-1}q_{j}}{\left(c\sum_{j=1}^{M} \eta_{ij}\kappa_{ij}^{-1}q_{j}\right)^{n}+\left(c\sum_{j=1}^{M} \kappa_{ij}^{-1}q_{j}\right)^{n}}$$

$$\frac{f_{i}(c)}{f_{\max}^{i}}\approx\left(c\sum_{j=1}^{M} \eta_{ij}\kappa_{ij}^{-1}q_{j}\right)^{n}+O(C^{2n})$$

Those glomerular channels activated at the lowest mixture concentration to invoke a coherent percept constitute the primacy set of ORs. To elicit a downstream response in projection neurons, the firing rate of an OSN must exceed a threshold $\theta{}_{i}$. Dropping the second-order terms in concentration, the relationship between affinity, efficacy, and concentration at the threshold is given by:

$$\left(C\sum_{j=1}^{M} \eta_{ij}\kappa_{ij}^{-1}q_{j}\right)^{n}=\theta{}_{i}$$

$$K_{ij}\equiv\eta_{ij}\kappa_{ij}^{-1}$$

$$c\sum_{j=1}^{M} K_{ij}q_{j}=\theta_{i}^{1/n}$$

Equation (S7) is of the same form as Equation , which was used in the main text to determine primacy sets. This suggests that our simple linear-nonlinear model can be used to determine primacy sets, even though OR activations are non-linear and include mixture interactions. This observation is based on the fact that primacy sets are determined at low odorant concentrations, at which the structure of OR response non-linearities is relatively simple and can be described by a single affinity matrix .

The experimental literature documents cases in which Hill coefficients i) do not significantly vary across ORs or OR-ligand pairs[[6](#_ENREF_6)], ii) vary across ORs[[7](#_ENREF_7)], and iii) vary across odors for the same OR[[7](#_ENREF_7), [8](#_ENREF_8)]. In this latter case, where the Hill coefficient may be specific to each OR-ligand pair (ie. $n_{ij}$), one theoretical study[[9](#_ENREF_9)] suggests that the dose-response curve of an OR-ligand mixture may be relatively invariant to manipulation of individual component Hill coefficients when compared to the other model parameters, at least in binary mixtures. We therefore assume equation (S1) to be a good approximation to equation (S7) and sufficient to define the primacy set for an odorant mixture.

This approximation tells us that the higher the value of $K_{ij}$, the lower the mixture concentration at which the response of OR $j$meets the threshold for evoking a downstream response and relaying partial information about the odorant. If the relevant space in which to consider the evolution of the OR repertoire was $K$-space in the main text, we propose that within this framing of receptor responses, ORs instead evolve in $A$-space. For a fixed threshold $\theta{}_{i}$, the product of affinity and efficacy defines the coordinates of an OR in this space. Assuming a primacy code for the computation of odor identity and a given environmental odor statistics, individual ORs will evolve to balance their affinities and efficacies to a subset of the physicochemical properties of interest to the olfactory system. This balance will involve maximizing the product of these variables subject to constraints imposed by the combinatorial nature of the primacy code, which requires ORs to respond with similar frequencies to the odor environment for an optimal code capacity[[10](#_ENREF_10)]. We suggest that evolutionary optimization via e.g. a birth-death model [[11](#_ENREF_11)] will drive the OR repertoire to be distributed as a primacy hull in $A$-space over an evolutionary timescale.

**Methods E. Formatting connectivity and affinity data: *FlyEM and FAFB*:** Each uPN-KC connectivity matrix $C_{PN}$ was first reduced to a glomerulus-KC connectivity matrix $C_{GL}$ by considering each glomerulus label in turn, summing all uPN connectivity vectors (rows in $C_{PN}$) associated with that glomerulus and adding the resulting vector as a row in the binary matrix $C_{GL}$.  *List of olfactory glomeruli:* D, DA1, DA2, DA3, DA4l, DA4m, DC1, DC2, DC3, DC4, DL1, DL2d, DL2v, DL3, DL4, DL5, DM1, DM2, DM3, DM4, DM5, DM6, DP1l, DP1m, V, VA1d, VA1v, VA2, VA3, VA4, VA5, VA6, VA7l, VA7m, VC1, VC2, VC3, VC4, VC5, VL1, VL2a, VL2p, VM1, VM2, VM3, VM4, VM5d, VM5v, VM6, VM7d, VM7v. A reduced version of this matrix was used for the analyses, shown in Fig.5, with OR labels corresponding to those in the DoOR affinity dataset.  *List of subset of glomeruli:* D, DA3, DA4l, DA4m, DC1, DC2, DC3, DL1, DL3, DL5, DM1, DM2, DM4, DM6, DP1l, VA1d, VA1v, VA2, VA3, VA4, VA5, VA6, VA7l, VC2, VC4, VC5, VL1, VL2a, VL2p, VM1, VM2, VM3, VM4, VM5d, VM5v, VM7d, VM7v, with the corresponding ORs determined as shown in Figure S4 and listed below.

***DoOR OR-odorant affinity:*** We extracted a dense subset of affinity scores from the DoOR dataset[[12](#_ENREF_12)]. This incomplete matrix contains affinities, which take values in the interval [0, 1], for 3488 OR-odorant pairs (60% complete).  The resulting matrix is of dimension 37 (ORs) x 156 (odorants).  We selected ORs i) that have a clear cognate glomerulus, i.e., all inputs to the glomerulus come from OSNs exclusively expressing that OR, and ii) for which there is sufficient data to distinguish relatively high affinities and define approximate primacy sets, i.e. ORs for which there are at least 18 $(2\max\left( p \right)+2)$ odorant affinities recorded. *List of glomeruli in dense subset of DoOR:* Or69a, Or23a, Or43a, Or2a, Or19a, Or13a, Or83c, Or10a, Or65a, Or7a, Or42b, Or22a, Or59b, Or67a, Ir75a, Or88a, Or47b, Or92a, Or67b, Or85d, Or49b, Or82a, Or46a, Or71a, Or67c, Ir41a, Ir75d, Ir84a, Ir31a, Ir92a, Or43b, Or9a, Ir76a, Or85b, Or98a, Or42a, Or59c.

**Methods F. Comparing FlyEM and FAFB datasets along the PN dimension via Monte Carlo alignment:**

To compare the binarized PN-KC connectivity matrices from the FlyEM and FAFB datasets ($W_{PN, KC}^{FlyEM}$ and $W_{PN, KC}^{FAFB}$ respectively), we aligned them along the unlabeled KC dimension as follows. First, we computed a distance matrix between the KCs belonging to different datasets $D_{FlyEM,FAFB}$. For each pair of the KCs, the distance between them was defined as one-half of the squared count of mismatching inputs from the PNs minus the squared count of matching inputs from the PNs:

$$D_{ij}={\frac{1}{2}\left( \sum_{k\in PNs} W_{ki}^{FlyEM}\left( {1-W}_{kj}^{FAFB} \right) \right)}^{2}-\left( \sum_{k\in PNs} W_{ki}^{FlyEM}W_{kj}^{FAFB} \right)^{2}.$$

This way, the matches were prioritized compared to the mismatches, reflecting the bias in the data annotation process where only the most certain projections were included in connectivity matrices. We then coarsely aligned the KCs of the two datasets. To do that, we initialized an empty matrix $W_{PN, KC}^{*}$ of a size of the larger (FlyEM) dataset. Then, on every iteration, we selected a random KC $i$ with no repetitions from the larger (FlyEM) dataset and used the distance matrix $D$ to determine the most similar KC $j$ from the smaller (FAFB) dataset: $j=\underset{j}{\mathrm{argmin}} D_{ij}$. The projection pattern $W_{PN, j}^{FAFB}$ of the KC $j$ was then removed from the smaller (FAFB) dataset and pasted to the matrix $W_{PN, KC}^{*}$ at the position $i$ matching that of the selected KC from the larger (FlyEM) dataset: $W_{PN, i}^{*}=W_{PN, j}^{FAFB}$. The process repeated until no KCs remained in the smaller (FAFB) dataset. Finally, to improve the match between the larger dataset $W_{PN, KC}^{FlyEM}$ and the coarsely aligned smaller dataset $W_{PN, KC}^{*}$, we permuted the latter along the KC dimension using a greedy Monte Carlo algorithm. Specifically, on every iteration, we permuted two random KCs $i,j$ in the coarsely aligned smaller dataset $W_{PN, KC}^{*}$ and computed the resulting change in distances: ${\Delta E=D}_{ii}+D_{jj}-D_{ij}-D_{ji}$. If this change $\Delta E$ was less than zero, we accepted the permutation; otherwise, we rejected it. We performed a total of ${10}^{7}$ iterations of the algorithm. To evaluate the alignment, we computed the fraction $f$ of synapses in the aligned smaller (FAFB) dataset matching the synapses of the larger (FlyEM) dataset: $W_{ij}^{*}=W_{ij}^{FlyEM}$. We evaluated the algorithm 10 times for the (same) data matrices and also 10 times for the (different) null models where both marginals were preserved. We then compared the alignment qualities $f$ for the data and null models using the paired-sample t-test with a threshold p-value of ${10}^{-3}$.

**Methods G. Comparing FlyEM and FAFB datasets along the OR dimension by analysis of correlations:**

To compare the binarized PN-KC connectivity matrices from the FlyEM and FAFB datasets regardless of the KC order, we performed the correlation analysis as follows. For each PN-KC connectivity matrix, we computed a matrix of Pearson correlations between the PN projection patterns: $W_{PN,KC}\to C_{PN,PN}$ where $C_{ij}=corr\left( W_{i*},W_{j*} \right)$. We then computed the Pearson correlations between these matrices for different datasets: $\mathfrak{C=}corr\left( C^{FlyEM},C^{FAFB} \right)$. We performed this procedure 10 times for (different) bootstrapped versions of the two datasets (where the KCs in each dataset were selected randomly with repetitions) and also 10 times for the (different) null models where both marginals were preserved. We then compared the correlation coefficients $\mathfrak{C}$ for the bootstrapped data and null models using the paired-sample t-test with a threshold p-value of ${10}^{-3}$.

**Methods H. Comparing FlyEM and FAFB datasets along the OR dimension via dimensionality reduction:**

To analyze the dimensionality of the binarized PN-KC connectivity matrices from the FlyEM and FAFB datasets, we used the dimensionality reduction techniques as follows. First, we normalized the binary connectivity matrices by shifting the means of every PN projection pattern to zero and scaling their variances to one. To obtain linear embeddings of the data, we performed the principal component analysis (PCA) on normalized projection matrices and computed the per-dimension variance of the PCA representation. To obtain non-linear embeddings of the data, we performed Isomap on normalized projection matrices and computed the per-dimension variance in the Isomap space in the same way as the variance computed in the previous step. To determine the optimal number of nearest neighbors to be used in the Isomap algorithm, we performed Isomap with the numbers or nearest neighbors from the minimum of 2 to the maximum of 50 using both FlyEM and FAFB datasets. For each number of nearest neighbors $k$, we computed the L2 norm of the difference $\Delta$ between Isomap representations of FlyEM and FAFB considering only the two first Isomap dimensions. We determined the optimal number of Isomap dimensions ($k=6$) by locating the elbow of the $\Delta\left( k \right)$ plot where the difference $\Delta$ began saturating as a function of the number of nearest neighbors $k$. We performed PCA and Isomap procedures for FlyEM and FAFB datasets, as well as for the corresponding (different) null models, 10 for each dataset. For the null models, we computed the mean and the variance of the pre-dimension variance explained.

**Methods I. Analysis of KC overlaps (Figure 5):** To compare the overlaps between OR-KC connectivity data and primacy sets contained in the OR-odorant affinity data, we first compute the overlap matrix (Fig. 5F) via the product $O(p)=AS(p)$. Each entry $O(p)_{ko}$represents the overlap between two binary vectors – one the OR inputs to KC number $k$, $A_{k,.}$, and the other, the primacy set corresponding to the odorant $o$, $S(p)_{.,o}$. For a given primacy number $p$, the matrix elements contained in$O(p)$ can be summarized as a histogram. First, we identified the overlaps for the ‘grandmother’ KC for each smell: the strength of connectivity of the highest overlapping KC with the corresponding primacy set. This strength can be combined in the vector $h(p,1)_{o}$, which contains the amount of overlap for odor $o$ between its primacy set and the best, ‘grandmother’ KC, for each primacy number. The histogram of this vector is shown in Figure 5F (minus the null model). Next, we computed the strengths of overlap for $l$ topmost KCs ($l=1$) for the case of ‘grandmother’. This yielded the quantity $h(p,1)_{o}$. This quantity is a tensor, which, for each odorant $o$, and a primacy number $p$, contains $l$ topmost overlaps. The histograms of values of this object are shown in Figures 5G and H, with the null model subtracted.

We compare the empirical distribution of overlaps contained in $h(p,1)_{o}$to the null model in which correlations are removed from the connectivity data by shuffling, we repeat the above analysis for each shuffled connectivity matrix, yielding an ensemble of matrices. We report the difference between empirical and null distributions via the difference matrix. Positive values (shown in red in Fig. 5F-H) indicate that there are more overlaps of a given degree between the empirical connectivity and primacy matrices, corresponding to sub-simplexes or faces of primary simplexes, relative to the null distribution. Negative values (shown in blue in Fig. 5F-H) indicate that the data contains fewer overlaps than would be expected under our null model. Statistical significance is assessed by computing an FDR-corrected p-value for each tuple [(p, overlap)].

**Methods J. Estimation of the effects of missing values in the DoOR dataset.**

A large fraction of the affinity matrix from the DoOR dataset is not known. To evaluate the effect of missing values on our conclusions, we generated an artificial dataset with similar characteristics and analyzed the consequences of removing values on our conclusions. First, we computed an estimated covariance matrix for the incomplete DoOR data, ignoring missing entries. We use this covariance matrix to generate a dense data matrix, Z, of size ($N_{odors}$+ $N_{KC}$) x$N_{OR}$, that has the same OR-OR covariance structure as the DoOR dataset. We used $N_{odors}$= 156, $N_{KC}$= 1750, $N_{OR}$= 37 as in the actual data. We took the first $N_{odors}$ rows of Z to be the surrogate complete affinity data. To construct related connectivity data, we took the last $N_{KC}$ rows of Z and binarized the data by setting the highest 7 entries in each row to 1 and all other entries to 0 (corresponding to a primacy number of 7). We increased the sparsity of this surrogate data to match that of the hemibrain connectivity by randomly selecting entries and setting them to 0. Both surrogate data matrices are thus related via the same primacy hull. We imposed the same missing structure on the surrogate affinity data as observed in the empirical DoOR data and observed that the proposed overlap test can indeed detect the shared primacy hull. Our analysis is thus sensitive to the structure in the data despite a substantial number of missing values.

**References**

1. Inagaki, S., et al., *Widespread Inhibition, Antagonism, and Synergy in Mouse Olfactory Sensory Neurons In Vivo.* Cell Reports, 2020. **31**(13): p. 107814.

2. Pfister, P., et al., *Odorant Receptor Inhibition Is Fundamental to Odor Encoding.* Current Biology, 2020. **30**(13): p. 2574-2587.e6.

3. Reddy, G., et al., *Antagonism in olfactory receptor neurons and its implications for the perception of odor mixtures.* eLife, 2018. **7**: p. e34958.

4. Singh, V., et al., *Competitive binding predicts nonlinear responses of olfactory receptors to complex mixtures.* Proc Natl Acad Sci U S A, 2019. **116**(19): p. 9598-9603.

5. Zak, J.D., et al., *Antagonistic odor interactions in olfactory sensory neurons are widespread in freely breathing mice.* Nature Communications, 2020. **11**(1): p. 3350.

6. Si, G., et al., *Structured Odorant Response Patterns across a Complete Olfactory Receptor Neuron Population.* Neuron, 2019. **101**(5): p. 950-962.e7.

7. Wachowiak, M. and L.B. Cohen, *Representation of Odorants by Receptor Neuron Input to the Mouse Olfactory Bulb.* Neuron, 2001. **32**(4): p. 723-735.

8. Firestein, S., C. Picco, and A. Menini, *The relation between stimulus and response in olfactory receptor cells of the tiger salamander.* The Journal of Physiology, 1993. **468**(1): p. 1-10.

9. Marasco, A., A. De Paris, and M. Migliore, *Predicting the response of olfactory sensory neurons to odor mixtures from single odor response.* Scientific Reports, 2016. **6**(1): p. 24091.

10. Zwicker, D., *Primacy coding facilitates effective odor discrimination when receptor sensitivities are tuned.* PLOS Computational Biology, 2019. **15**(7): p. e1007188.

11. Andersson, M.N., C. Löfstedt, and R.D. Newcomb, *Insect olfaction and the evolution of receptor tuning.* Frontiers in Ecology and Evolution, 2015. **3**.

12. Münch, D.a.G., C. Giovanni, *DoOR 2.0 - Comprehensive Mapping of Drosophila melanogaster Odorant Responses,* Scientific Reports, 2016. **6**.

13. Munch, D., *ODOR REPRESENTATIONS IN DROSOPHILA RECEPTOR NEURONS ANALYZED BY IN VIVO CALCIUM IMAGING*, in *Mathematisch-Naturwissenschaftliche Sektion Fachbereich Biologie*. 2014, UNIVERSITÄT KONSTANZ.
