# Supplementary material for: The primacy model and the structure of olfactory space
Source: PLoS Comput Biol. 2024 Sep 10;20(9):e1012379. doi: 10.1371/journal.pcbi.1012379 (PMC11423968; doi:10.1371/journal.pcbi.1012379)
Supplement: S1 Fig — (A) Glomeruli placed in the connectivity’s 2D PCA space. The same glomeruli in two datasets are connected by lines. Glomeruli are colored according to their function as indicated. The first PC of connectivity appears to be related to food-sensitive glomeruli, while the second PC is unrelated to food. (B-D) Three first PCs for the two datasets plotted against each other. The first two PCs (B and C) are conserved between FlyEM and FAFB datasets (R = 0.92 and 0.78), while the third PC appears to be random. This indicates that only the first two PCs of connectivity are conserved across individual animals. (E-H) For randomly shuffled connectivity matrices, none of the principal components are conserved across individuals. (PDF) [file pcbi.1012379.s002.pdf]

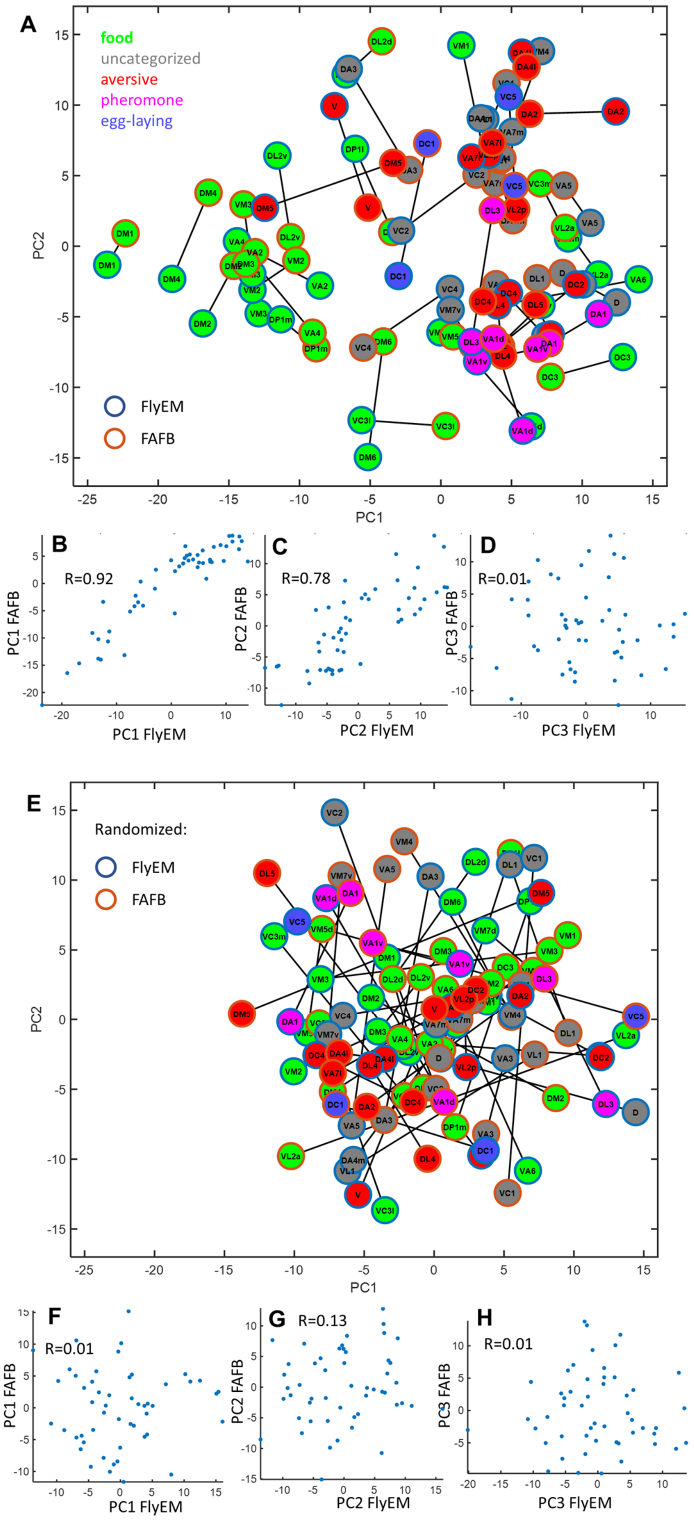

S1 Fig. Functional significance of non-random features of connectivity in FlyEM and FAFB datasets. (A) Glomeruli placed in the connectivity's 2D PCA space. The same glomeruli in two datasets are connected by lines. Glomeruli are colored according to their function as indicated. The first PC of connectivity appears to be related to food-sensitive glomeruli, while the second PC is unrelated to food. (B-D) Three first PCs for the two datasets plotted against each other. The first two PCs (B and C) are conserved between FlyEM and FAFB datasets (R=0.92 and 0.78), while the third PC appears to be random. This indicates that only the first two PCs of connectivity are conserved across individual animals. (E-H) For randomly shuffled connectivity matrices, none of the principal components are conserved across individuals.
