# Supplementary material for: The primacy model and the structure of olfactory space
Source: PLoS Comput Biol. 2024 Sep 10;20(9):e1012379. doi: 10.1371/journal.pcbi.1012379 (PMC11423968; doi:10.1371/journal.pcbi.1012379)
Supplement: S2 Fig — Instead, they are related to the connectivity structure. (A) Binarized connectivity matrix in FlyEM dataset with both ORs and KCs sorted according to their contribution to the first PC (PC1). ORs with similar PC1 appear to have stronger connectivity, suggesting that the structure of OR-KC connections determines the contribution of ORs to a PC. (B) The number of connections made by KC in the binarized matrix does not have a clear monotonic dependence on PC1. Thus, the first PC is not produced by differences in the KC in-degree. (C, D) Same for PC2. A diagonal band along the diagonal in the sorted connectivity matrices in (A) and (C) indicates that ORs are connected to specific groups of KC, which determines both PC1s. Thus PC1 and PC2 emerge from a specific connectivity structure. (E-H) The same analysis in the randomized matrices shows that the first PC is correlated with the KC in-degree (F). (PDF) [file pcbi.1012379.s003.pdf]

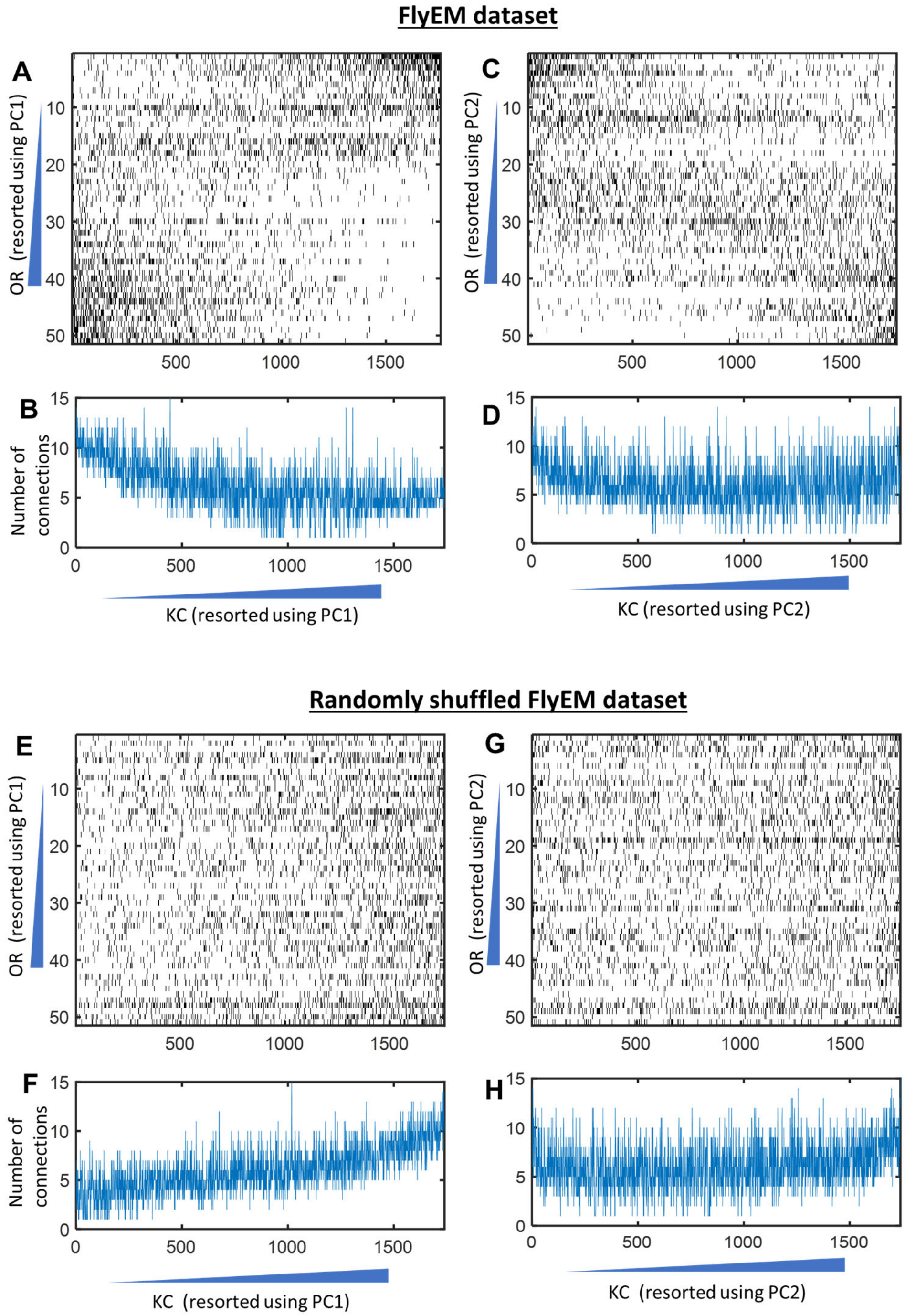

S2 Fig. The first two PCs of connectivity cannot be explained by the in-degree of the KCs. Instead, they are related to the connectivity structure. (A) Binarized connectivity matrix in FlyEM dataset with both ORs and KCs sorted according to their contribution to the first PC (PC1). ORs with similar PC1 appear to have stronger connectivity, suggesting that the structure of OR-KC connections determines the contribution of ORs to a PC. (B) The number of connections made by KC in the binarized matrix does not have a clear monotonic dependence on PC1. Thus, the first PC is not produced by differences in the KC in-degree. (C, D) Same for PC2. A diagonal band along the diagonal in the sorted connectivity matrices in (A) and (C) indicates that ORs are connected to specific groups of KC, which determines both PC1s. Thus PC1 and PC2 emerge from a specific connectivity structure. (E-H) The same analysis in the randomized matrices shows that the first PC is correlated with the KC in-degree (F).
