# Supplementary material for: The primacy model and the structure of olfactory space
Source: PLoS Comput Biol. 2024 Sep 10;20(9):e1012379. doi: 10.1371/journal.pcbi.1012379 (PMC11423968; doi:10.1371/journal.pcbi.1012379)
Supplement: S3 Fig — (A) Two connectivity datasets aligned using the simulated annealing algorithm. (B) Same for two randomly shuffled connectivity matrices. (C) Even randomly shuffled connectivity matrices share ~77% of synapses, when aligned. Unshuffled connectivity matrices share ~78% synapses. (D) Hamming distances between aligned KCs. Only 16 KCs are an exact match (H = 0) versus 6 KCs in the randomly shuffled case. (PDF) [file pcbi.1012379.s004.pdf]

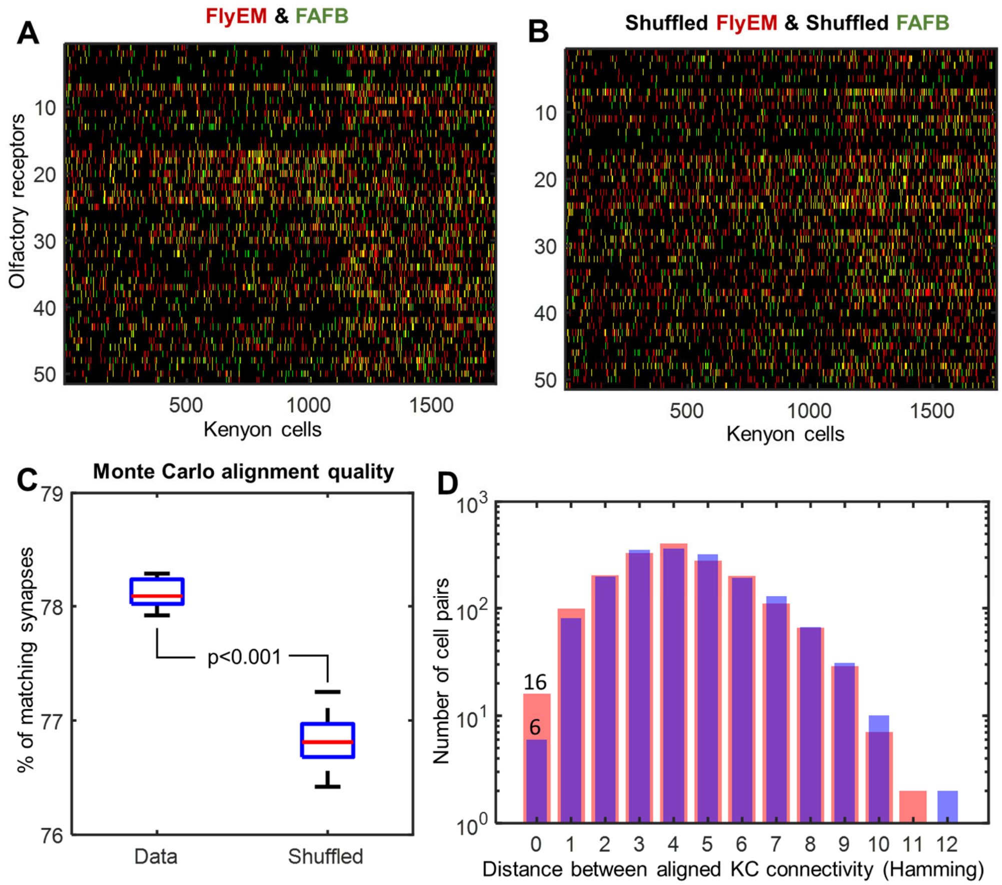

S3 Fig. Results of brute force alignment of connectivity datasets. (A) Two connectivity datasets aligned using the simulated annealing algorithm. (B) Same for two randomly shuffled connectivity matrices. (C) Even randomly shuffled connectivity matrices share ~77% of synapses, when aligned. Unshuffled connectivity matrices share ~78% synapses. (D) Hamming distances between aligned KCs. Only 16 KCs are an exact match (H=0) versus 6 KCs in the randomly shuffled case.
