# Supplementary material for: The primacy model and the structure of olfactory space
Source: PLoS Comput Biol. 2024 Sep 10;20(9):e1012379. doi: 10.1371/journal.pcbi.1012379 (PMC11423968; doi:10.1371/journal.pcbi.1012379)
Supplement: S4 Fig — Each glomerulus in the Drosophila antennal lobe receives input from specific OR types. The majority receive input from only a single odorant-responsive receptor type, however some glomeruli, e.g. DL1, receive converging input from >1 OR type. Left: OR types present in the DoOR dataset are represented by a filled green circle, while a blue circle represents those ORs missing from the DoOR. Right: Glomeruli that are included in Figs 4 and S1 are labeled in bold and those that are included in our analysis of DoOR data (Fig 5) are labeled in bold red. Based on data from [13] (S1 Text). (PDF) [file pcbi.1012379.s005.pdf]

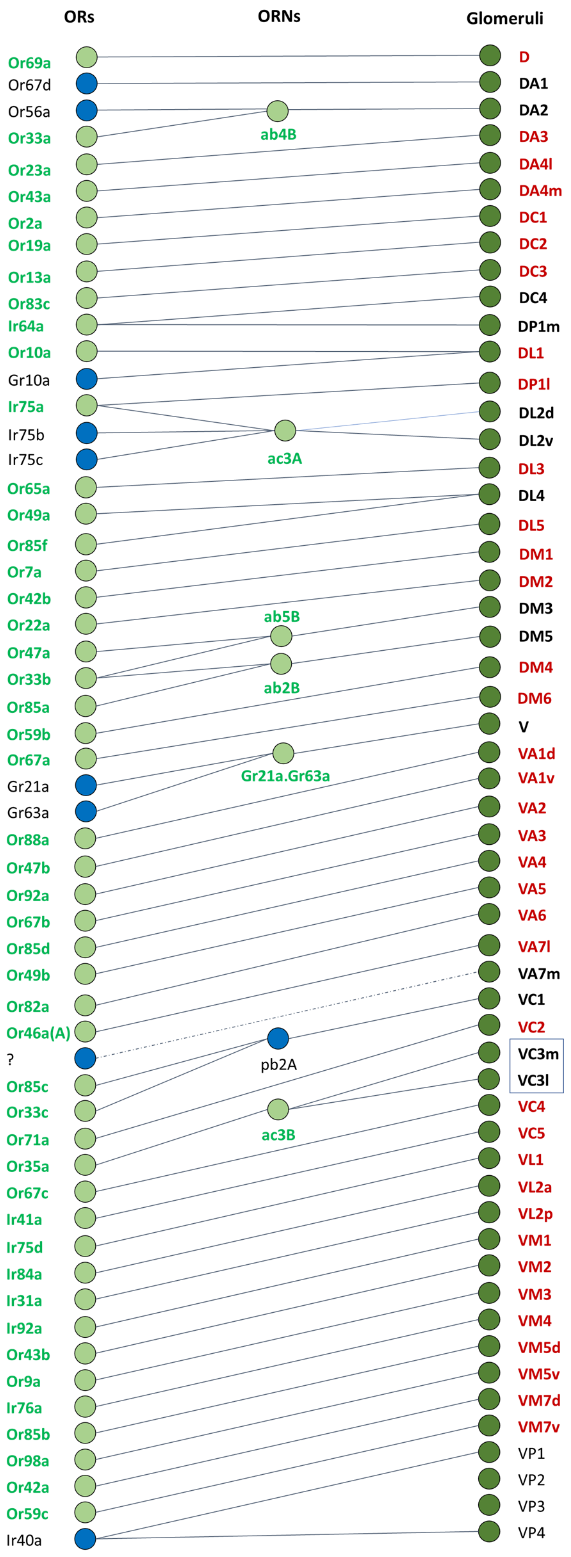

S4 Fig. Glomeruli and their cognate odorant receptors. Each glomerulus in the *Drosophila* antennal lobe receives input from specific OR types. The majority receive input from only a single odorant-responsive receptor type, however some glomeruli, e.g. DL1, receive converging input from >1 OR type. *Left:* OR types present in the DoOR dataset are represented by a filled green circle, while a blue circle represents those ORs missing from the DoOR. *Right:* Glomeruli that are included in Figures 4 and S1 are labeled in bold and those that are included in our analysis of DoOR data (Figure 5) are labeled in bold red. Based on data from [13] (S1 Text).
