# Supplementary material for: The primacy model and the structure of olfactory space
Source: PLoS Comput Biol. 2024 Sep 10;20(9):e1012379. doi: 10.1371/journal.pcbi.1012379 (PMC11423968; doi:10.1371/journal.pcbi.1012379)
Supplement: S5 Fig — Left: OR-OR Pearson correlation matrices for FlyEM and FAFB connectivity data (37 glomeruli). Right: OR-OR Pearson correlation matrices for primacy sets in DoOR affinity data shown for a range of primacy numbers. Correlations between connectivity and primacy sets are reported for FlyEM (in purple) and FAFB (in green). Statistically significant correlation coefficients are recorded for a range of primacy numbers. (PDF) [file pcbi.1012379.s006.pdf]

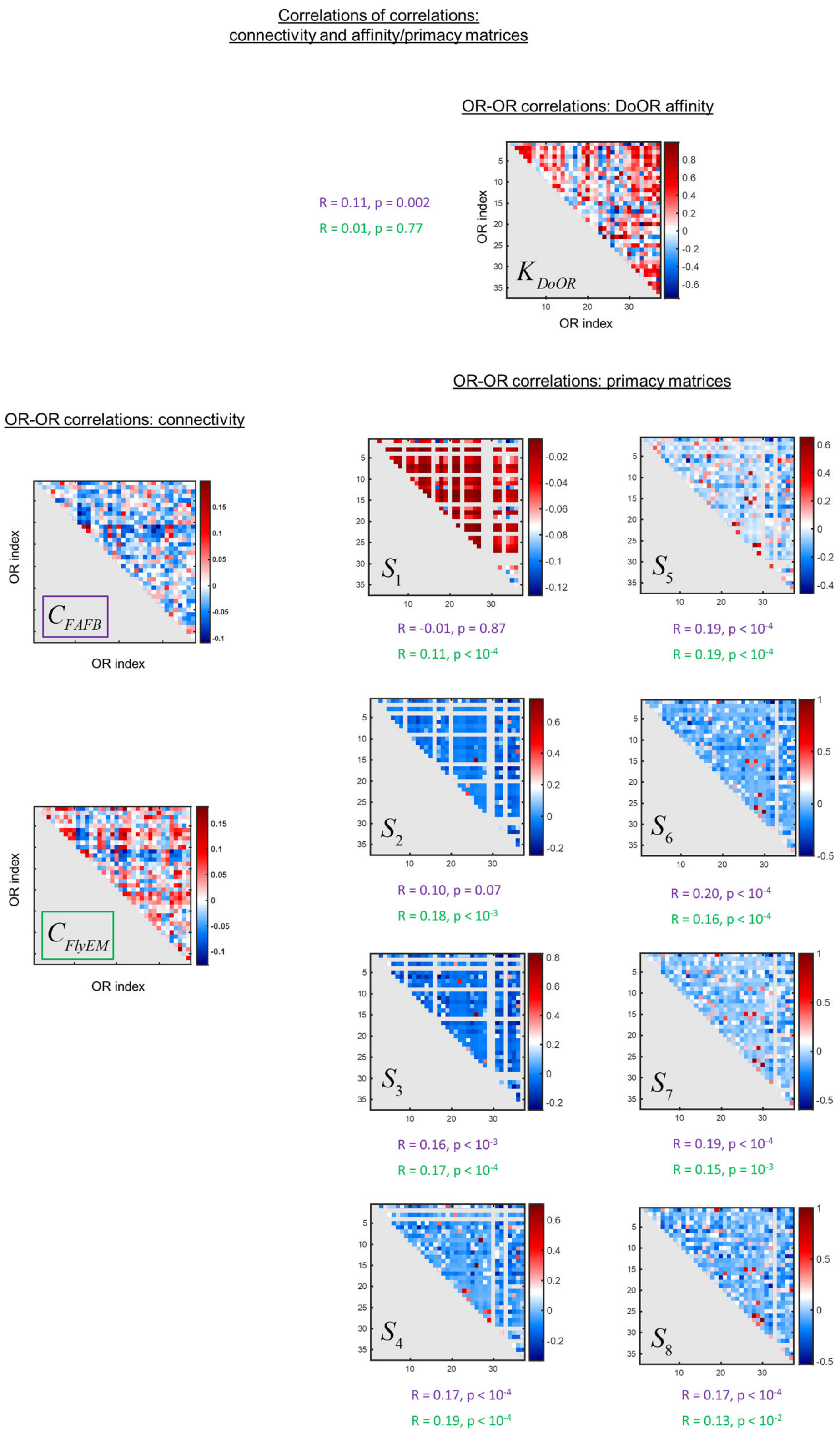

S5 Fig. *Left:* OR-OR Pearson correlation matrices for FlyEM and FAFB connectivity data (37 glomeruli). *Right*: OR-OR Pearson correlation matrices for primacy sets in DoOR affinity data shown for a range of primacy numbers. Correlations between connectivity and primacy sets are reported for FlyEM (in purple) and FAFB (in green). Statistically significant correlation coefficients are recorded for a range of primacy numbers.
