# Supplementary material for: The primacy model and the structure of olfactory space
Source: PLoS Comput Biol. 2024 Sep 10;20(9):e1012379. doi: 10.1371/journal.pcbi.1012379 (PMC11423968; doi:10.1371/journal.pcbi.1012379)
Supplement: S6 Fig — We first generate i) a surrogate affinity matrix with similar statistical properties to the DoOR dataset and ii) a related surrogate connectivity dataset. Both datasets are related via the same primacy hull (see Section S10 above). We impose the same missing structure on the surrogate affinity data as observed in the empirical DoOR data and observe that the proposed overlap test can indeed detect the shared primacy hull. (PDF) [file pcbi.1012379.s007.pdf]

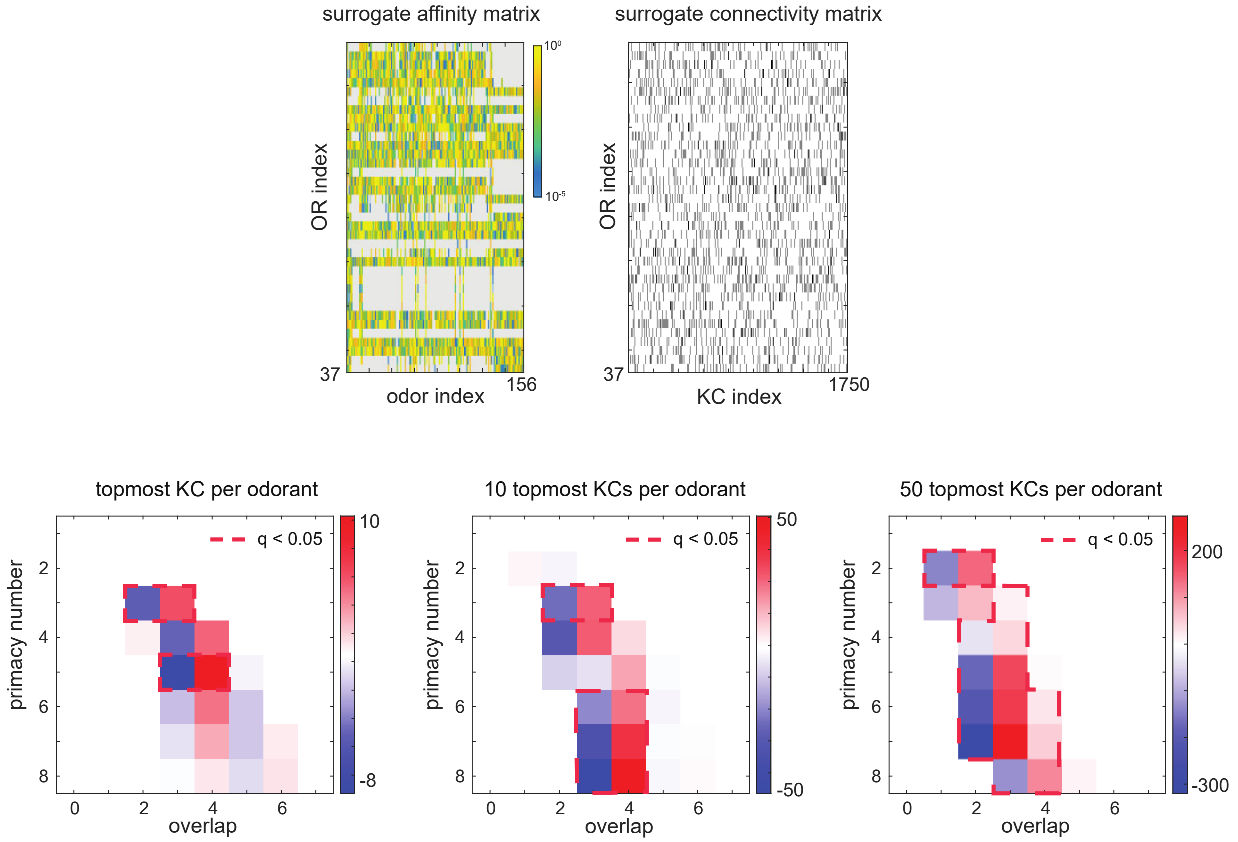

S6 Fig. To investigate the sensitivity of the test applied in Fig. 5 of the main text to missing data, we first generate i) a surrogate affinity matrix with similar statistical properties to the DoOR dataset and ii) a related surrogate connectivity dataset. Both datasets are related via the same primacy hull (see Section S10 above). We impose the same missing structure on the surrogate affinity data as observed in the empirical DoOR data and observe that the proposed overlap test can indeed detect the shared primacy hull.
